# A Coordinated Mitochondrial Genome Expression Program Marks a Differentiated Neural-Lineage and Synaptic Transcriptional State in Lower-Grade Glioma

**DOI:** 10.3390/ijms27167289

**Published:** 2026-08-15

**Authors:** Henrique Ritter Dal-Pizzol, Mariane Jaeger, Caroline Brunetto de Farias, André T. Brunetto, Gustavo R. Isolan, Rafael Roesler

**Affiliations:** 1 Department of Pharmacology, Institute for Basic Health Sciences, Federal University of Rio Grande do Sul, Porto Alegre 90035-003, Brazil; hpizzol@hcpa.edu.br; 2 Cancer and Neurobiology Laboratory, Experimental Research Center, Clinical Hospital (CPE-HCPA), Federal University of Rio Grande do Sul, Porto Alegre 90035-003, Brazil; 3 National Science and Technology Institute for Children’s Cancer Biology and Pediatric Oncology—INCT BioOncoPed, Porto Alegre 90035-003, Brazil; 4 Children’s Cancer Institute (ICI), Porto Alegre 90620-110, Brazil; 5 Graduate Program in Biological Sciences: Pharmacology and Therapeutics, Institute for Basic Health Sciences, Federal University of Rio Grande do Sul, Porto Alegre 90035-003, Brazil; 6 Graduate Program in Principles of Surgery, Mackenzie Evangelical University, Curitiba 80730-000, Brazil; 7 The Center for Advanced Neurology and Neurosurgery (CEANNE), Porto Alegre 90560-010, Brazil; 8 Center for Biotechnology, Federal University of Rio Grande do Sul, Porto Alegre 91501-970, Brazil

**Keywords:** mitochondrial DNA, mtPCG, mitochondria, oxidative phosphorylation, lower-grade glioma, glioma, brain tumor

## Abstract

Mitochondria regulate cellular metabolism, signaling, and cell fate, yet the biological significance of coordinated mitochondrial genome transcription in lower-grade glioma (LGG) remains unclear. We integrated transcriptomic and clinical data from the TCGA and CGGA cohorts to investigate expression of the 13 mitochondrial DNA-encoded protein-coding genes (mtPCGs). Elevated expression of all mtPCGs was consistently associated with favorable molecular subtypes and prolonged overall survival (OS). Transcriptome-wide correlation and Gene Ontology analyses revealed a coordinated program enriched for synaptic signaling, neurotransmission, postsynaptic organization, and neuronal differentiation, accompanied by positive correlations with neuronal and synaptic markers and negative correlations with stemness and immune-associated markers. Although the mitochondrial transcriptional score was not independently associated with survival after adjustment for established clinicomolecular variables, it consistently marked biologically less aggressive tumors. Coordinated mitochondrial genome expression identifies a neuron-like transcriptional state associated with favorable LGG biology, revealing an unexpected link between mitochondrial transcription, synaptic identity, and glioma differentiation.

## 1. Introduction

**Figure 1:**
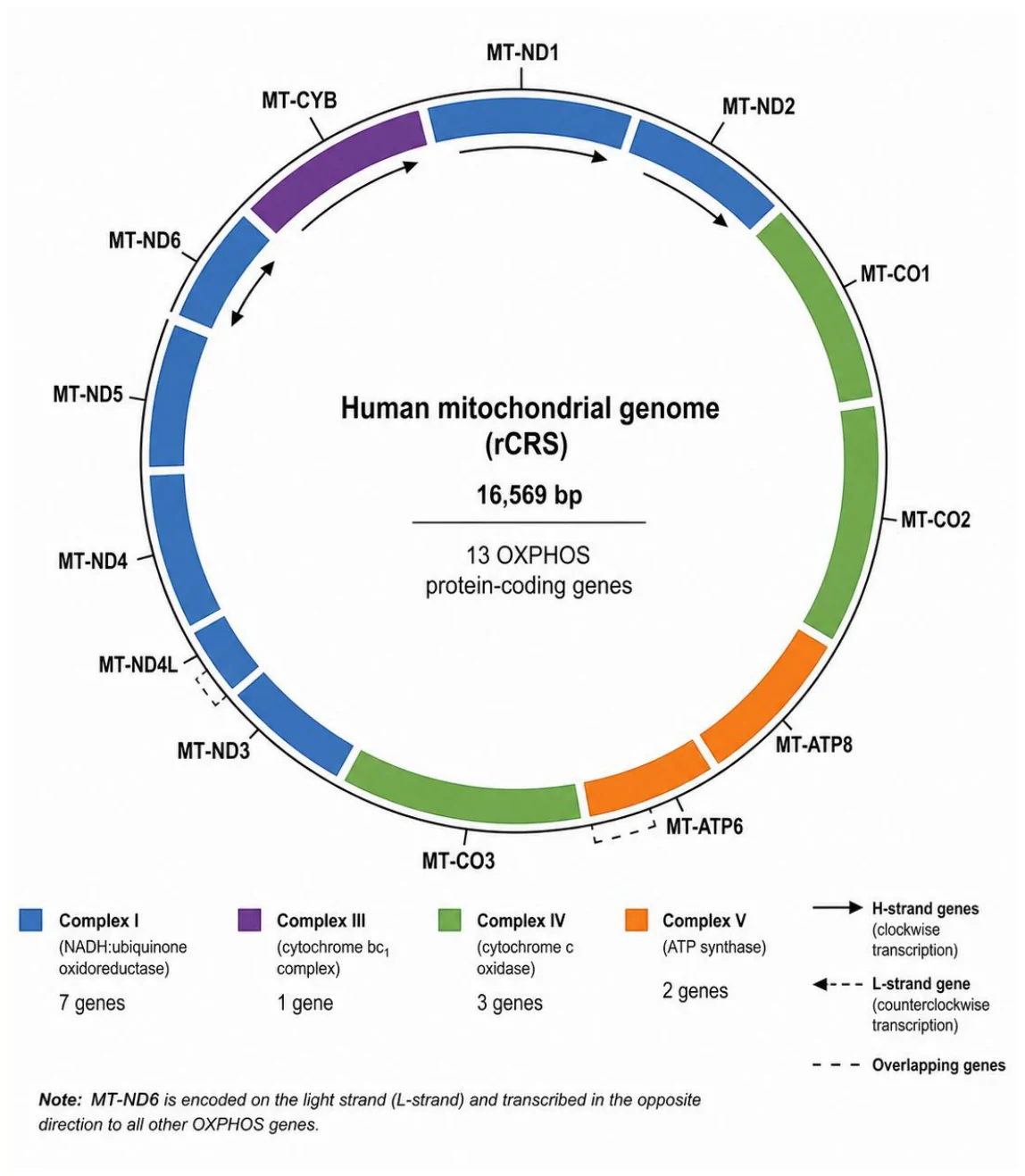
Schematic representation of the 13 mtPCGs in the human circular mitochondrial genome.

Mitochondria perform numerous functions beyond ATP production, including regulation of cellular signaling, differentiation, and cell death through mechanisms involving reactive oxygen species (ROS), modulation of calcium homeostasis, release of mitochondrial proteins that activate apoptosis, and retrograde communication with the nucleus [1,2,3]. Mitochondria contain a semi-autonomous, self-replicating genome composed of mitochondrial DNA (mtDNA). Human mtDNA is organized as a compact circular genome of 16,569 base pairs (bp) that contains 13 essential protein-coding genes collectively encoding core components of the oxidative phosphorylation (OXPHOS) system, the principal machinery responsible for aerobic ATP production. In genomic order, these mitochondrial protein-coding genes (mtPCGs) are *MT-ND1*, *MT-ND2*, *MT-CO1*, *MT-CO2*, *MT-ATP8*, *MT-ATP6*, *MT-CO3*, *MT-ND3*, *MT-ND4L*, *MT-ND4*, *MT-ND5*, *MT-ND6*, and *MT-CYB*. Their protein products are incorporated into mitochondrial respiratory chain complexes I, III, IV, and V, where they participate in electron transport, proton gradient generation, and ATP synthesis [4,5,6] (Figure 1).

Altered mitochondrial function and dysregulated OXPHOS activity have been implicated in multiple aspects of cancer biology. Different tumor types exhibit either increased or reduced mitochondrial content and OXPHOS activity [3,7,8], and mtDNA copy number has been associated with tumor stage in several solid malignancies. In addition, mtDNA mutations and inhibition of mitochondrial gene transcription may alter intracellular signaling pathways crucial in cancer, such as mitogen-activated protein kinase (MAPK) and phosphoinositide 3-kinase (PI3K)/AKT/mammalian target of rapamycin (mTOR) signaling [8,9].

Lower-grade gliomas (LGGs) comprise a heterogeneous group of diffusely infiltrating gliomas that include World Health Organization (WHO) grade 2 and grade 3 tumors. Contemporary classification integrates histopathological features with molecular markers, particularly isocitrate dehydrogenase (IDH) mutation status and whole-arm 1p/19q codeletion [10]. These molecular alterations define biologically distinct glioma subtypes with markedly different clinical behavior, treatment responses, and patient outcomes. In particular, IDH-mutant, 1p/19q-codeleted oligodendrogliomas generally exhibit the most favorable prognosis, whereas IDH-wildtype diffuse gliomas tend to display more aggressive clinical characteristics [11,12,13]. As a result, molecular classification has become a central component of glioma diagnosis, prognostication, and biological investigation.

Mitochondrial DNA represents a promising molecular platform for the development of diagnostic and prognostic biomarkers in cancer due to features such as its short length and relatively simple structure, which facilitate genomic analysis [8]. Therefore, characterizing the expression patterns and potential clinical associations of mtDNA-encoded genes in LGGs may help identify novel prognostic markers and therapeutic opportunities.

Here, we characterized the expression profiles, prognostic significance, and transcriptome-wide correlation patterns of the 13 mtPCGs in LGG. Our findings demonstrate that increased and coordinated mtPCG expression is associated not only with improved clinical outcomes but also with a distinct neuronal/synaptic transcriptional program enriched for genes involved in synaptic signaling, neuronal differentiation, and synaptic plasticity. These results identify coordinated mitochondrial transcription as a marker of a neural lineage-like state associated with less aggressive glioma biology.

## 2. Results

### 2.1. Expression of mtPCGs Across Different LGG Types

**Figure 2:**
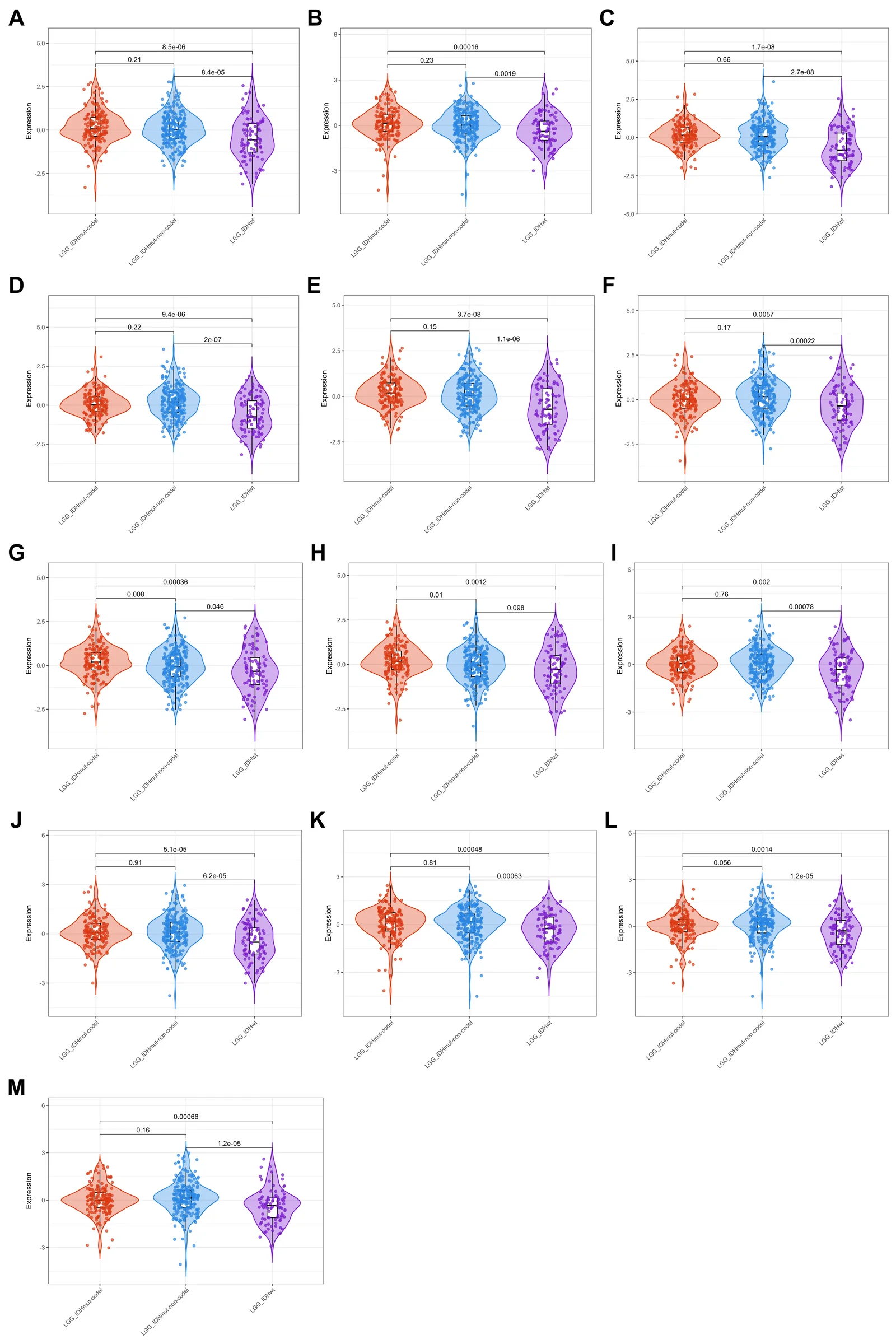
Gene expression levels of mtPCGs in TCGA LGG tumors classified into molecular subtypes. (**A**) *MT-ND1*; (**B**) *MT-ND2*; (**C**) *MT-CO1*; (**D**) *MT-CO2*; (**E**) *MT-ATP8*; (**F**) *MT-ATP6*; (**G**) *MT-CO3*; (**H**) *MT-ND3*; (**I**) *MT-ND4L*; (**J**) *MT-ND4*; (**K**) *MT-ND5*; (**L**) *MT-ND6*; (**M**) *MT-CYB*. LGG-IDH-mut-codel, *n* = 164, LGG-IDH-mut-non-codel, *n* = 235, LGG-IDH-wt, *n* = 91; *p*-values are indicated in the panels.

We first investigated mtPCG expression patterns in LGG tumors according to either histological classification or molecular subtype. When the Cancer Genome Atlas (TCGA) [14] and Chinese Glioma Genome Atlas (CGGA) [15] tumors were stratified by histological type, no substantial overall differences in expression profiles were observed among groups, although several individual comparisons reached statistical significance (Supplementary Figures S1 and S2). Analyses based on molecular classification revealed a clearer expression pattern, characterized by the highest mtPCG expression levels in LGG harboring IDH mutation with 1p/19q co-deletion (LGG-IDH-mut-codel), the lowest levels in IDH wild-type tumors (LGG-IDH-wt), and intermediate expression in IDH-mutant tumors lacking 1p/19q co-deletion (LGG-IDH-mut-non-codel), in both the TCGA (Figure 2) and CGGA (Supplementary Figure S3) datasets.

### 2.2. High mtPCG Expression Is Associated with Longer OS in Patients with LGG

**Figure 3:**
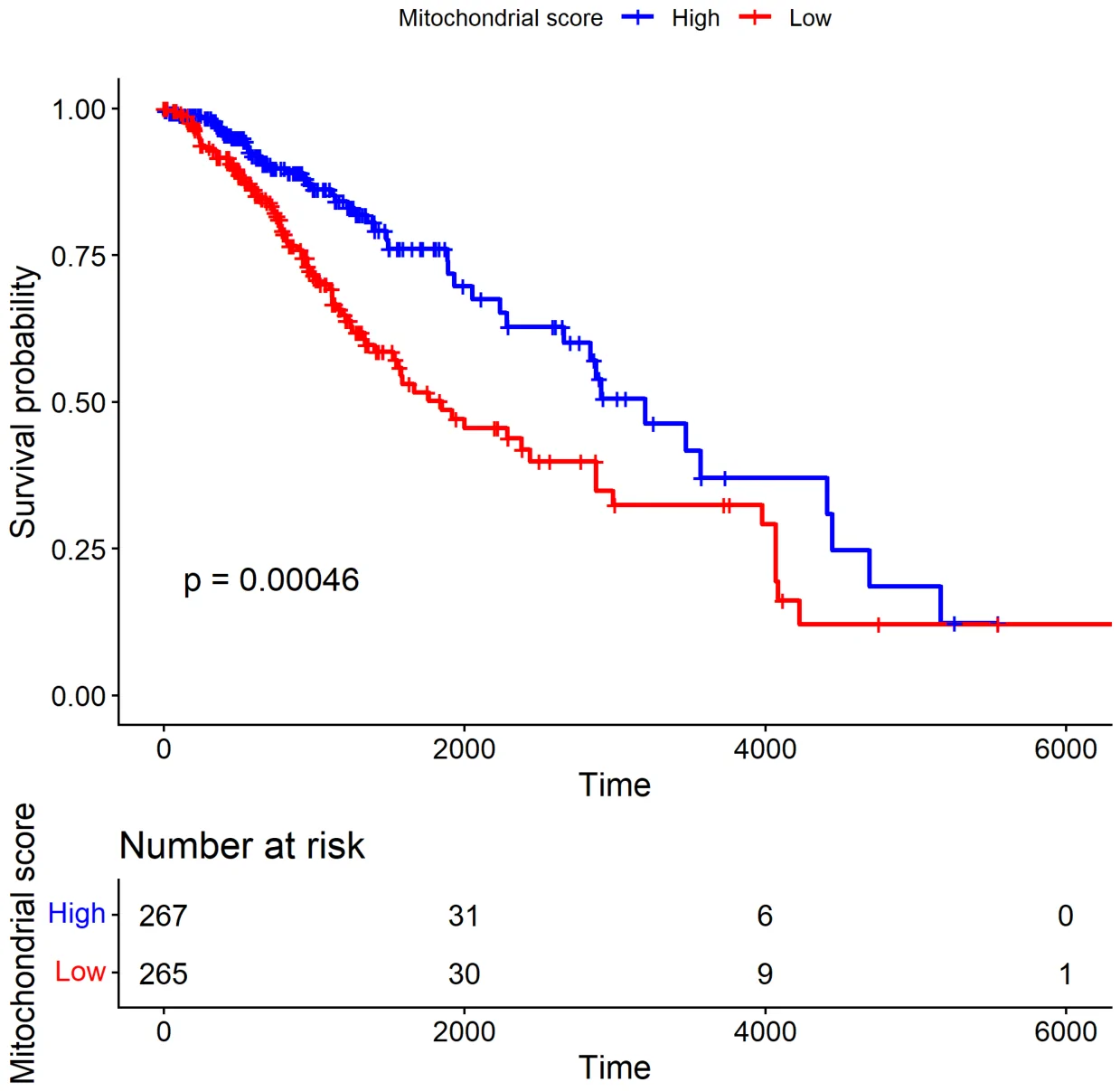
High gene expression of a composite mitochondrial score from the combined expression levels of all 13 mtPCGs is associated with longer survival in patients with TCGA LGG tumors. The number of samples and *p*-value are indicated in the panels.

To verify whether expression of mtDNA-encoded genes is related to patient prognosis, we performed Kaplan–Meier analyses of OS comparing patients with LGG tumors divided into low- or high-expressing groups. We found that high expression of all of the 13 mtPCGs was significantly associated with longer overall survival (OS) in both the TCGA (Supplementary Figure S4) and CGGA (Supplementary Figure S5) datasets. A significant association between the mitochondrial gene score and patient survival was also observed when transcription levels of all 13 mtPCGs were combined into a “mitochondrial gene score” (Figure 3).

To determine whether the mitochondrial transcriptional signature exerted prognostic effects independent of established molecular determinants of LGG outcome, multivariate Cox regression analyses were performed including age, tumor grade, IDH status, 1p/19q codeletion status, and the mitochondrial gene score. Although the mitochondrial score showed a strong univariate association with OS, its confidence intervals crossed the null hazard ratio; therefore, this association did not remain statistically significant after adjustment for the established molecular prognostic factors (Supplementary Figure S6). These findings indicate that the prognostic value of the mitochondrial transcriptional signature is not independent of established molecular determinants of outcome and likely reflects its close association with biologically favorable glioma subtypes.

### 2.3. Correlations Among Expression of mtPCGs

**Figure 4:**
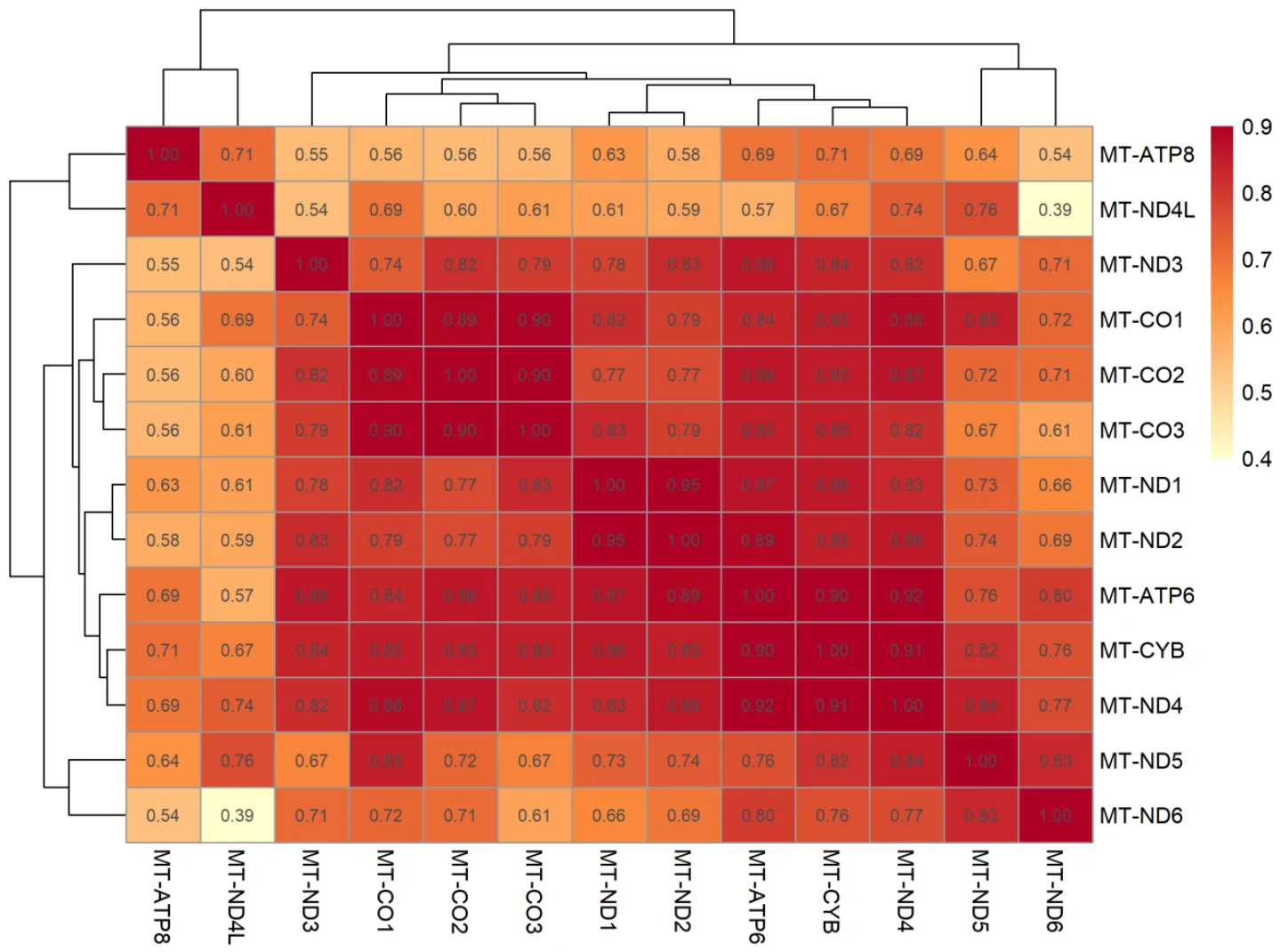
Co-expression structure of mtPCG expression in LGG. Pairwise correlations among the 13 mtPCGs were computed in TCGA LGG using RNA-seq data processed via Genomic Data Commons (GDC)/TCGAbiolinks. Expression values were log2-transformed and z-scored across samples. Spearman correlation coefficients were calculated for all gene pairs to generate a gene–gene correlation matrix. Genes were hierarchically clustered using Ward’s method and visualized as a heatmap (pheatmap, R (version 4.5.1)). The color scale is indicated in the figure.

We then evaluated correlations among expression of the different mtPCGs. Moderate to strong positive correlations were found among most genes in two different analyses, suggesting a coordinated gene expression program (Figure 4 and Figure S7; Supplementary Table S1).

### 2.4. Functional Characterization of the Mitochondrial Transcriptional Program in LGG

**Figure 5:**
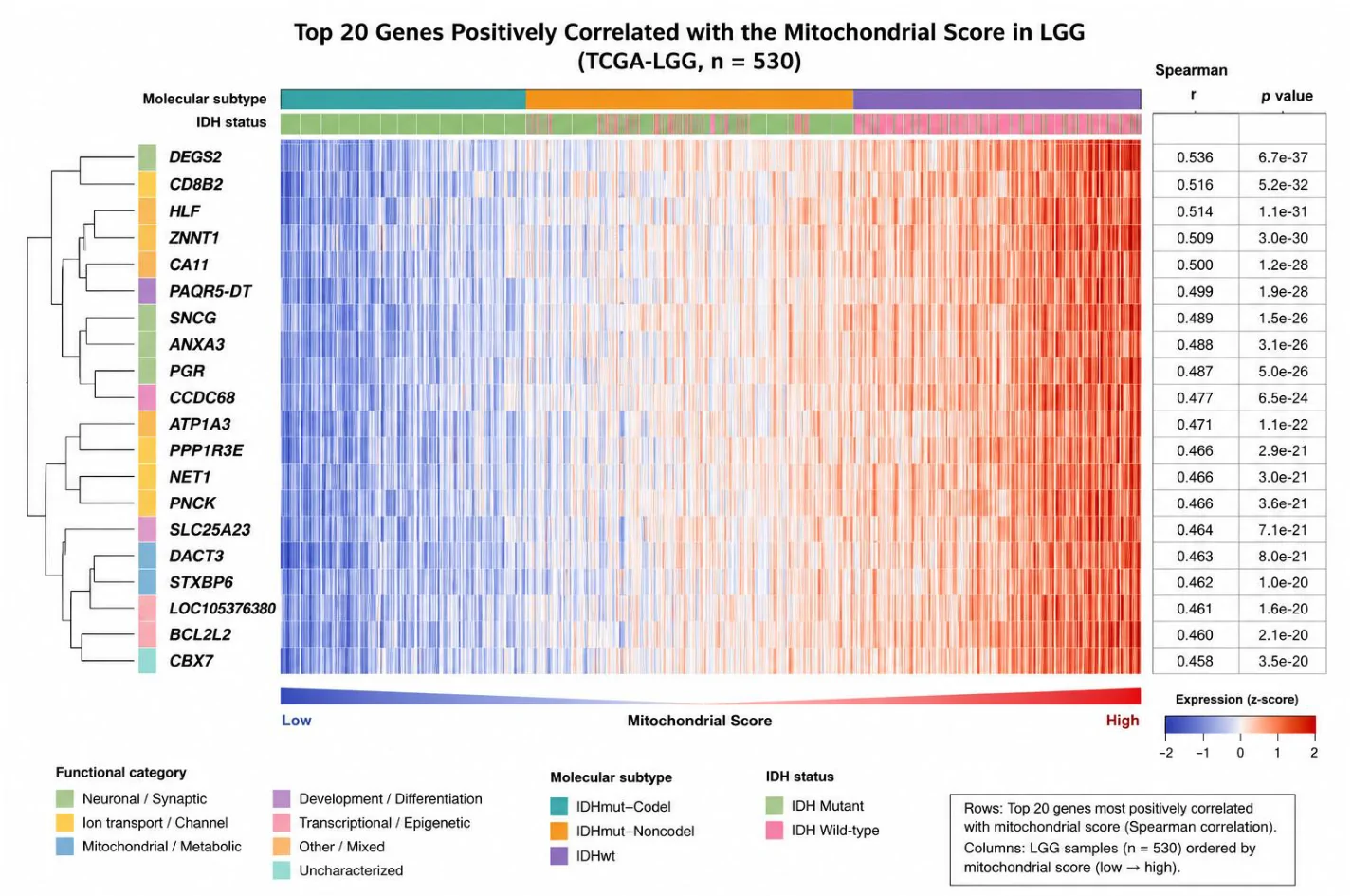
Correlation matrix displaying the twenty genes showing the strongest positive correlations with the mitochondrial score in TCGA-LGG tumors. Colors represent Spearman correlation coefficients, with darker shades indicating stronger positive correlations. The analysis identifies a group of genes related to neuronal identity, synaptic structure, and neurotransmission that exhibit coordinated expression with mitochondrial protein-coding genes.

To explore the biological significance of coordinated mitochondrial genome expression in LGG, unbiased transcriptome-wide correlation analyses were performed using the mitochondrial score derived from the expression of the 13 mtPCGs. Heatmap visualization of the top correlated genes demonstrated a coordinated transcriptional program associated with increasing mitochondrial score values. Functional enrichment analyses of the top 200 genes most strongly associated with the mitochondrial score highlighted a prominent enrichment of neuronal and synaptic processes (Figure 5). The most significantly enriched Gene Ontology (GO) Biological Process terms included chemical synaptic transmission and regulation of neuronal synaptic plasticity. Additional enriched processes included clustering of voltage-gated sodium channels and retrograde neuronal dense-core vesicle transport. GO Molecular Function analyses further highlighted channel activity and Soluble N-ethylmaleimide-sensitive factor Attachment protein REceptor (SNARE) binding, supporting an association with neuronal signaling and vesicular trafficking. Several genes positively correlated with the mitochondrial score were involved in synaptic transmission and neuronal function, including *ATP1A3*, *VAMP2*, *SHANK1*, *KCNN1*, *SLC8A2*, and *CBLN2* (Supplementary Figure S8).

### 2.5. Association of the Mitochondrial Score with Cell Lineage Markers

**Figure 6:**
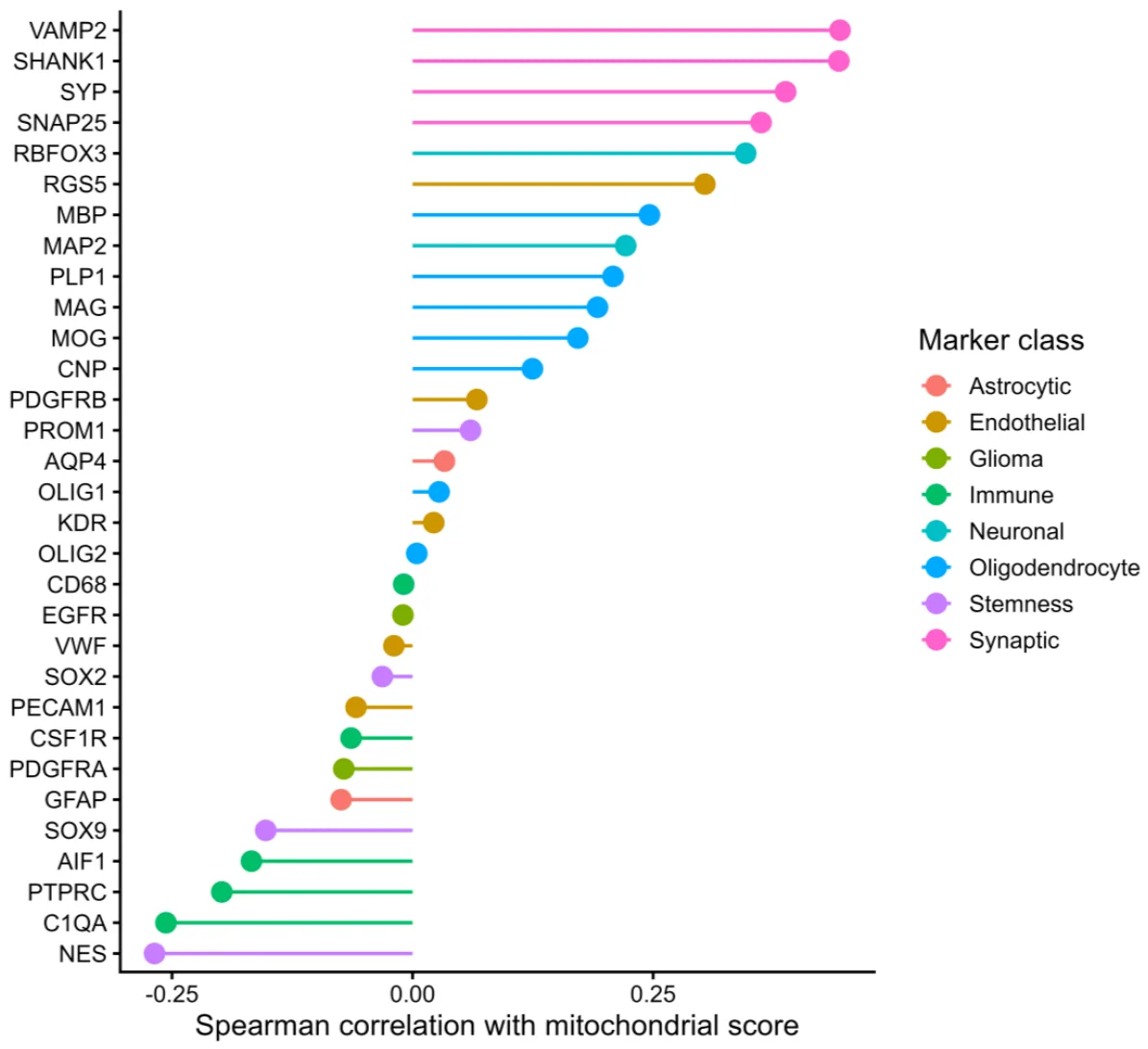
Association of the mitochondrial score with representative cell lineage markers in LGG. Spearman correlation coefficients between the mitochondrial score and representative markers of neuronal, synaptic, oligodendrocyte-lineage, astrocytic, immune, endothelial, glioma-associated, and stemness-related cellular states. Positive correlations were observed primarily with neuronal and synaptic markers (*VAMP2*, *SHANK1*, *SYP*, *SNAP25*, *RBFOX3*) and oligodendrocyte-lineage markers (*MBP*, *PLP1*, *MAG*, *MOG*, *CNP*), whereas immune-associated markers (*C1QA*, *PTPRC*, *AIF1*), stemness markers (*NES*, *SOX9*, *SOX2*), and several vascular markers (*PECAM1*, *VWF*) showed negative correlations. Colors indicate marker classes.

To further investigate the cellular context of this transcriptional signature, correlations between the mitochondrial score and representative lineage markers were examined. The mitochondrial score showed positive correlations with neuronal and synaptic markers, including *VAMP2* (*r* = 0.44), *SHANK1* (*r* = 0.44), *SYP* (*r* = 0.39), *SNAP25* (*r* = 0.36), and *RBFOX3* (*r* = 0.35). Positive correlations were also observed with oligodendrocyte-lineage markers, including *MBP* (*r* = 0.25), *PLP1* (*r* = 0.21), *MAG* (*r* = 0.19), and *MOG* (*r* = 0.17). In contrast, immune markers such as *C1QA* (*r* = −0.26), *PTPRC* (*r* = −0.20), and *AIF1* (*r* = −0.17), as well as stemness-associated markers including *NES* (*r* = −0.27) and *SOX9* (*r* = −0.15), showed negative correlations with the mitochondrial score (Figure 6).

## 3. Discussion

Large-scale cancer genomics initiatives such as TCGA and CGGA have generated extensive transcriptomic datasets from large cohorts of glioma patients, enabling comprehensive analyses of mitochondrial gene expression patterns in human tumors [9,14,15,16]. Using this strategy, we identified a consistent transcriptional landscape in which increased expression of the 13 mtPCGs, both individually and as a combined gene set, was strongly associated with improved survival in patients with LGG. Importantly, our results do not support the interpretation that mtPCG expression constitutes an independent prognostic biomarker in LGG. Rather, the loss of statistical significance after adjustment for IDH status and 1p/19q codeletion suggests that elevated mitochondrial gene expression is closely linked to molecular states already known to confer favorable clinical outcomes. Therefore, the mitochondrial transcriptional program identified here is best interpreted as a feature of a broader biological phenotype associated with tumor differentiation and reduced aggressiveness, rather than as a prognostic factor operating independently of established molecular classifications. Specifically, the highest expression in LGG-IDH-mut-codel tumors supports the view that mtPCGs are associated with a more favorable biological prognosis. Also, the present findings should be interpreted as evidence of association rather than causation; our analyses do not establish whether mitochondrial gene expression actively contributes to these biological features or merely reflects underlying transcriptional states characteristic of less aggressive tumors. The present study focused specifically on the 13 mtDNA-encoded protein-coding genes. Future investigations integrating mitochondrial genome expression with nuclear-encoded mitochondrial genes, mtDNA copy number, mtDNA mutational profiles, and other indicators of mitochondrial function may provide a more comprehensive understanding of mitochondrial states associated with LGG biology and clinical outcome.

Early reports of elevated mRNA expression of specific mtDNA-encoded genes in cancer cells [7,17,18,19] were followed by a growing body of work investigating the role of mitochondrial respiratory chain components and mitochondria-associated genes in tumor progression and malignancy [3,7,8]. Recently, an analysis of 515 lung adenocarcinoma samples from the TCGA and Gene Expression Omnibus (GEO) databases demonstrated that patients could be stratified according to mitochondrial-related gene expression profiles, with the subgroup exhibiting enhanced mitochondrial metabolic activity showing a poorer clinical prognosis [20].

Glioma cells show an oxidative metabolic phenotype where mitochondrial respiration is crucial for energy production, and targeting the OXPHOS system has been explored as a potential therapeutic strategy [21]. In GBM cells, depletion of the mitochondria-associated methyltransferase METTL17, which stimulates OXPHOS through mitochondrial RNA methylation, impairs cell proliferation, migration, and invasion, and *METTL17* overexpression reversed these effects [22]. In addition, high expression of genes coding glycolytic enzymes, *HK2* and *PKM2*, and low expression of genes involved in mitochondrial oxidative metabolism, *SDHB* and *COX5A*, were associated with poorer GBM patient survival [23]. Characterization of tumor cell states and GBM subtypes based upon neurodevelopmental and metabolic axes identified a mitochondrial GBM phenotype that relied exclusively on oxidative phosphorylation for energy production and was associated with favorable clinical outcome [24]. Copy number alterations in mitochondrial DNA can also influence GBM tumorigenesis [25].

Recent studies have increasingly highlighted the relevance of mitochondrial biology in LGG, although from perspectives distinct from the present work. LGG tumors can display disrupted mitochondrial functions and an increased number of mutations in mitochondrial genome coding genes, possibly related to increased oxidative stress. Variations in mtDNA sequences in these cells may lead to protein malfunction and OXPHOS impairment [26]. A 9-gene signature composed of mitochondrial-related genes other than mtPCGs, *ACOT7*, *THEM5*, *MTHFD2*, *ABCB7*, *PICK1*, *PDK3*, *ARMCX6*, *GSTK1*, and *SSBP1*, consistently stratified glioma patients into high- and low-risk groups in the TCGA and CGGA cohorts [27]. A study investigating associations between germline mtDNA variants and glioma risk and mortality identified common variants m.3010G>A, m.195T>C, and m.16189T>C as being associated with LGG risk, although these associations did not remain statistically significant after correction for multiple testing [28]. Using TCGA and CGGA RNA-seq datasets, Yang et al. [29] identified 12 prognosis-related mitochondrial-related genes (MRGs) and developed a mitochondrial activation score associated with poorer OS in LGG. Similarly, Zhou et al. [30] proposed a mitochondrial RNA modification (MRM) score that predicted malignant clinical characteristics, immune infiltration, genomic alterations, pathway enrichment, metabolism, and clinical outcome, while also identifying patients more likely to respond to immunotherapy and targeted therapies. The study by Li et al. [31] identified a mitochondrial-related gene cluster significantly associated with poor prognosis, whereas Mou et al. [10] analyzed approximately 200 oxidative phosphorylation-related genes and identified two molecular subtypes of LGG, with the C2 subtype exhibiting significantly worse survival. These studies support the importance of mitochondrial biology in LGG but primarily focus on nuclear-encoded mitochondrial genes, mitochondrial signaling pathways, or mitochondrial functional states.

In contrast to these previous studies, the present report specifically investigates the transcriptional program of the 13 mtPCGs, representing the mitochondrial genome itself rather than nuclear mitochondrial regulators. Furthermore, our findings extend beyond prognostic associations by demonstrating that coordinated mtPCG expression is consistently associated with favorable clinical and molecular features and, importantly, with a neural lineage marker profile and a synaptic transcriptional signature. We have recently begun exploring the hypothesis that increased expression of genes associated with excitatory synaptic signaling and synaptic plasticity might contribute to a more favorable clinical behavior by conferring a more mature, neuronal-like phenotype to LGG tumors. Genes encoding α-amino-3-hydroxy-5-methyl-4-isoxazolepropionic acid (AMPA) glutamate receptor (AMPAR) subunits are associated with a synaptic-enriched transcriptional program in LGG [32]. High expression of *DLG2*, *DLG3*, and *DLG4*, which encode synaptic scaffolding proteins of the membrane-associated guanylate kinase (MAGUK) family, PSD-93 (SAP-93), SAP-102, and PSD-95, respectively, was strongly correlated with the expression of a synaptic gene signature and was associated with significantly longer survival in both the TCGA and CGGA LGG cohorts [33]. The mitochondrial OXPHOS system is increasingly recognized as an integral component of the synaptic and neuronal plasticity machinery [34,35,36,37,38]. Synaptic transmission and activity-dependent plasticity are highly dependent on mitochondrial function, and pharmacological or metabolic inhibition of mitochondrial activity has been shown to impair synaptic signaling and long-term potentiation (LTP) [39,40,41,42]. Notably, neuronal activity associated with hippocampal LTP induction promotes increased expression of mitochondrial genes [43].

Within this framework, the association between elevated expression of the mitochondrial protein-coding genome and improved clinical outcome may reflect a biological program in which coordinated upregulation of genes involved in excitatory synaptic transmission, neuronal differentiation, and synaptic plasticity contributes to a less aggressive phenotype in LGGs. In fact, our functional enrichment analyses demonstrated that genes associated with the mitochondrial score were enriched not only for mitochondrial-related functions but also for neuronal and synaptic processes, including chemical synaptic transmission, regulation of neuronal synaptic plasticity, ion channel organization, and vesicular transport. These findings support the view that elevated mitochondrial gene expression may occur within a broader neuronal-like transcriptional state. This interpretation is biologically plausible given the substantial energetic demands associated with synaptic signaling, neurotransmitter release, ion transport, and neuronal plasticity, all of which require robust mitochondrial support.

**Figure 7:**
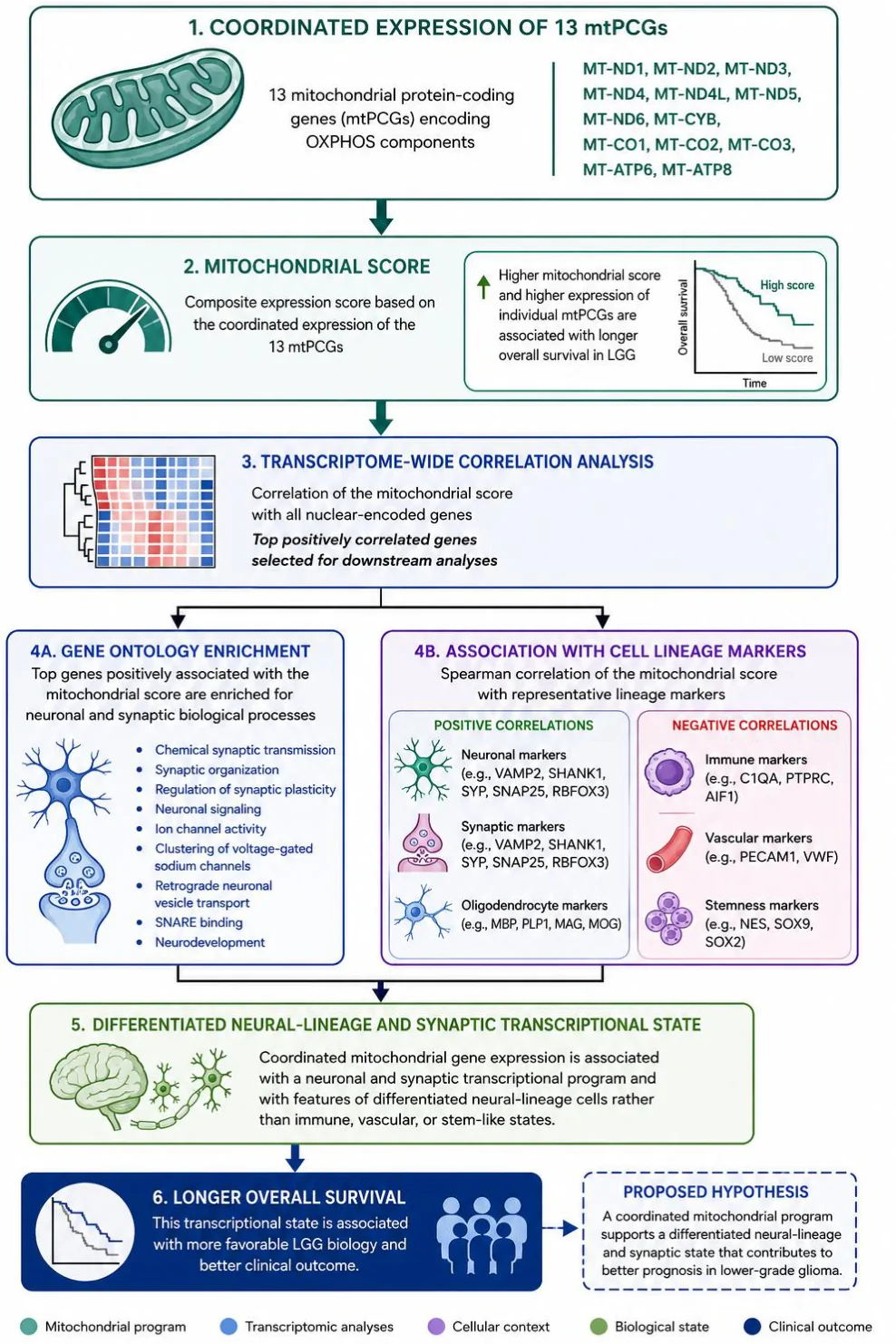
Proposed biological framework linking coordinated mitochondrial gene expression to a neural-lineage and synaptic transcriptional program in LGG. The 13 mtPCGs encoding OXPHOS components exhibit coordinated expression in LGG. Higher expression of individual mtPCGs and of the composite mitochondrial score is associated with longer patient OS. Transcriptome-wide correlation analyses identified a gene expression program enriched for neuronal and synaptic biological processes, including chemical synaptic transmission, synaptic organization, neuronal signaling, and ion channel activity. Correlation analyses with representative lineage markers further demonstrated positive associations with neuronal, synaptic, and oligodendrocyte-lineage markers and negative associations with immune, vascular, and stemness-related markers. Together, these findings suggest that coordinated mitochondrial gene expression is associated with a differentiated neural-lineage and synaptic transcriptional state linked to more favorable LGG biology and clinical outcome.

Further supporting this interpretation, the mitochondrial score correlated positively with multiple neuronal, synaptic, and oligodendrocyte-lineage markers while showing negative associations with immune, vascular, and stemness-associated markers. These observations suggest that the mitochondrial transcriptional program identified in bulk LGG transcriptomes is unlikely to be driven primarily by immune or vascular components of the tumor microenvironment. Instead, the data support an association with differentiated neural-lineage transcriptional states. Although bulk transcriptomic analyses do not allow definitive assignment of gene expression to specific cell populations, these results provide important biological context for the favorable prognostic associations observed in the present study. Collectively, the findings suggest that coordinated mtPCG expression in LGG may represent a marker of a more differentiated neural-like state characterized by enhanced synaptic and neuronal gene expression programs (Figure 7).

An important limitation of the present study is that transcriptomic associations with patient survival, as well as correlations among different gene expression signatures, should not be interpreted as evidence of direct biological causation. Rather, these findings are best viewed as a framework for the generation of mechanistic hypotheses and the identification of potential prognostic transcriptional states. Functional studies will be necessary to determine whether mtPCGs actively contribute to the modulation of LGG aggressiveness. Experimental approaches involving gene silencing or genetic manipulation in LGG models may help clarify whether mtPCGs exert direct tumor-suppressive effects.

Another relevant consideration is that mRNA abundance does not necessarily correlate with protein expression or functional protein activity. Although several studies across different biological systems have reported substantial concordance between transcriptomic and proteomic measurements, others have demonstrated only modest correlations between mRNA and protein levels [44,45,46]. Consequently, validation at the protein and functional levels will be important to further establish the biological significance of the transcriptional patterns identified in this study. Yet another limitation is that we did not evaluate the frequency of elevated expression of individual mtPCGs across patients or according to WHO grade. Our analyses focused on transcriptomic associations and prognostic implications at the cohort level, and future studies investigating the prevalence of mtPCG overexpression in specific clinicopathological subgroups may provide further insight into the heterogeneity of mitochondrial transcriptional programs in LGG.

Finally, the transcriptomic datasets analyzed in this study were generated from bulk tumor tissue. Consequently, the observed mtPCG expression patterns cannot be unequivocally attributed to specific cellular populations. Although the correlation analyses performed here provide evidence that the mitochondrial transcriptional program is associated with neuronal, synaptic, and oligodendrocyte-lineage gene expression states and is negatively associated with immune and vascular markers, these findings do not establish the precise cellular source of mtPCG expression. Therefore, the mitochondrial transcriptional signature identified here should be interpreted as a property of the tumor tissue as a whole rather than as a tumor-cell-specific program. Future studies employing single-cell RNA sequencing, spatial transcriptomics, or more advanced computational deconvolution approaches will be necessary to determine the cellular origin of mtPCG expression and its relationship to glioma biology.

## 4. Materials and Methods

### 4.1. Datasets and Gene Expression Analyses

RNA-sequencing data together with corresponding clinical annotations for patients with LGG were obtained from two independent cohorts comprising World Health Organization (WHO) grade 2 and grade 3 gliomas: The TCGA (https://www.cancer.gov/tcga, accessed on 13 August 2026) and CGGA (http://www.cgga.org.cn, accessed on 13 August 2026) datasets [14,15]. WHO grade 3 gliomas have historically been considered “high-grade” and similar to GBM in some clinical contexts; however, the present study used the TCGA-LGG and CGGA cohorts, which were specifically designed to include diffuse WHO grade 2 and grade 3 gliomas. Consistent with the nomenclature used by these datasets and by a large body of prior transcriptomic studies, we used the term “lower-grade glioma” (LGG) in this study. For the TCGA cohort, gene-level expected count data were retrieved from the TOIL repository through the UCSCXenaTools package (version 1.7.0; rOpenSci project, Auckland, New Zealand). Clinical information was obtained from the TCGA Pan-Cancer Atlas through the cBioPortalData package. For the CGGA cohort, RNA-sequencing and clinical datasets from the CGGA-325 and CGGA-693 projects were downloaded and integrated by retaining genes common to both datasets. To minimize potential technical variability between datasets, batch origin (CGGA-325 versus CGGA-693) was included as a covariate in the DESeq2 design formula during differential expression analyses. Samples lacking survival information or incomplete clinical annotations were excluded from downstream analyses.

TCGA expected counts were converted back to integer-compatible count values to enable count-based modeling with DESeq2. For CGGA, raw count matrices from both datasets were merged after harmonization of shared genes. When duplicate gene symbols were identified, the transcript displaying the highest average expression across samples was retained. Variance-stabilizing transformation was then applied to normalized counts using DESeq2, followed by gene-wise z-score standardization within each cohort.

Expression patterns of mtPCGs were initially examined according to the previous histopathological classification of gliomas [14,47], including astrocytoma (TCGA, *n* = 188; CGGA, *n* = 239), oligodendroglioma (TCGA, *n* = 179; CGGA, *n* = 145), and oligoastrocytoma (TCGA, *n* = 123; CGGA, *n* = 23). Analyses were subsequently performed incorporating the updated molecular classification system [48] in both TCGA and CGGA cohorts, consisting of LGG-IDH-mut-codel (TCGA, *n* = 164; CGGA, *n* = 113), LGG-IDH-mut-non-codel (TCGA, *n* = 235; CGGA, *n* = 142), and LGG-IDH-wt (TCGA, *n* = 91; CGGA, *n* = 88). Groupwise comparisons were performed using the Wilcoxon rank-sum test, and resulting *p*-values were corrected for multiple comparisons using the False Discovery Rate (FDR) Benjamini–Hochberg (BH) procedure.

Mitochondrial gene scores were calculated separately within each cohort after variance-stabilizing transformation and gene-wise z-score normalization. For each sample, the score was defined as the arithmetic mean of the standardized expression values of the 13 mtPCGs. All genes contributed equally to the score, and no weighting procedure was applied. The same methodology was used for both TCGA and CGGA datasets.

### 4.2. Survival Analysis

Associations between OS and expression levels of mtPCGs were investigated using Kaplan–Meier survival analyses. Patients were stratified into high- and low-expression groups according to optimal expression thresholds identified with the surv_cutpoint function from the survminer package (Supplementary Table S2). Differences between survival curves were assessed using the log-rank test, and *p*-values were adjusted for multiple testing across genes and cohorts using the FDR BH correction.

All statistical analyses were conducted in the R environment (version 4.5.1). RNA-sequencing count data were subjected to variance-stabilizing transformation and batch adjustment using the DESeq2 package (version 1.48.1) prior to downstream expression analyses. Survival analyses were carried out with the survival package (version 3.8.3), and Kaplan–Meier plots were generated using survminer. Data visualization was performed with ggplot2 (version 4.0.2). Data retrieval and preprocessing were conducted using UCSCXenaTools (version 1.7.0) and cBioPortalData (version 2.20.0).

### 4.3. Multivariate Survival

Multivariate Cox proportional hazards regression analyses were performed to evaluate whether the mitochondrial gene score in TCGA tumors was independently associated with OS after adjustment for established clinical and molecular prognostic variables. Covariates included age, tumor grade, IDH mutation status, and 1p/19q co-deletion status. Hazard ratios (HRs) and 95% confidence intervals (CIs) were calculated using the survival package in R.

### 4.4. Gene Expression Correlations

Co-expression relationships among mtPCGs were examined using both the Evergene platform (https://bshihlab.shinyapps.io/evergene/, accessed on 13 August 2026) [49] and analyses conducted within the R statistical environment. Pearson correlation was used for mtPCG–mtPCG co-expression analyses because these analyses were restricted to the 13 mPCG genes after normalization and standardization, with the specific aim of evaluating linear coordination within a compact, biologically defined mitochondrial gene set. Positive moderate correlations were defined as Pearson’s *r* between 0.45 and 0.61, strong correlations as *r* between 0.62 and 0.88, and very strong correlations as *r* > 0.89 with a statistically significant *p* value. Spearman rank correlation was used for transcriptome-wide gene-versus-mitochondrial-score analyses and for correlations with representative lineage markers because these comparisons involved a much larger and more heterogeneous set of genes, for which expression distributions may differ substantially and may include non-linear monotonic relationships and outlier-sensitive patterns. Spearman correlation was therefore selected as a more appropriate non-parametric approach that is robust to distributional heterogeneity across genes. For correlation analyses performed in R, gene expression values were standardized across samples through z-score normalization. Pairwise associations between genes were calculated using Spearman’s rank correlation coefficient in order to evaluate monotonic relationships without assuming normal data distribution. A symmetric correlation matrix was subsequently generated to represent gene co-expression patterns. Hierarchical clustering was performed using Ward’s minimum variance method, and heatmaps were produced with the pheatmap package in R. Correlation strength was represented by a color gradient ranging from blue (negative correlations) to red (positive correlations). Missing values were handled through pairwise deletion, and no data imputation procedures were applied.

### 4.5. Functional Characterization of the Mitochondrial Gene Expression Program

To investigate the biological processes associated with coordinated mitochondrial gene expression in LGG, we used the mitochondrial expression score calculated for each TCGA-LGG sample and the mean normalized expression of the 13 mitochondrial protein-coding genes. Gene-level RNA-sequencing data from the TCGA-LGG cohort were obtained from the UCSC Xena platform using the GDC STAR-transformed transcriptomic dataset. Spearman correlation coefficients were calculated between the mitochondrial score and the expression of all genes in the dataset. Genes were ranked according to correlation coefficients, and mitochondrial genes, mitochondrial pseudogenes, and transcripts lacking gene symbol annotation were excluded from downstream analyses. The 200 genes showing the strongest positive correlations with the mitochondrial score were selected for functional enrichment analyses.

Gene Ontology (GO) enrichment analyses were performed using the Enrichr platform (https://maayanlab.cloud/Enrichr/, accessed on 13 August 2026) [50], including Biological Process, Cellular Component, and Molecular Function categories. In addition, to further investigate the potential cellular context of the mitochondrial transcriptional program, correlations between the mitochondrial score and representative markers of neuronal/synaptic differentiation, oligodendrocyte lineage, stemness, immune cell infiltration, and vascular compartments were calculated using Spearman correlation analysis.

### 4.6. Use of Generative Artificial Intelligence

During preparation of this manuscript, OpenAI’s ChatGPT (GPT-5.5) was used to assist in the graphical design of Figure 1 and Figure 7 and to assist in the identification of grammar and language issues during the final revision of the manuscript. All data analyses, scientific interpretation, figure content, and final editing were performed and verified by the authors, who take full responsibility for the published work.

## 5. Conclusions

Our results demonstrate that coordinated expression of the 13 mtPCGs is consistently associated with favorable molecular subtypes and longer survival in LGG. Beyond its prognostic associations, the mitochondrial transcriptional program was linked to genes involved in neuronal and synaptic biological processes and showed positive correlations with neuronal, synaptic, and oligodendrocyte-lineage markers while correlating negatively with immune, vascular, and stemness-associated markers. Together, these findings suggest that elevated mitochondrial gene expression occurs within a broader differentiated neural-lineage and synaptic transcriptional state associated with more favorable LGG biology. Although the mitochondrial score was not independently prognostic after adjustment for established molecular determinants of outcome, its reproducible association with favorable clinicomolecular features highlights coordinated mitochondrial gene expression as a biologically informative component of LGG transcriptional heterogeneity.

## Data Availability

This research used data generated by The Cancer Genome Atlas (TCGA) Research Network (https://www.cancer.gov/tcga, accessed on 13 August 2026); the Chinese Glioma Genome Atlas (CGGA, http://www.cgga.org.cn, accessed on 13 August 2026), including data accessed and analyzed through the Evergene (https://bshihlab.shinyapps.io/evergene/, accessed on 13 August 2026) and Enrichr (https://maayanlab.cloud/Enrichr/, accessed on 13 August 2026) platforms.

## Supplementary Materials

The following supporting information can be downloaded at: https://www.mdpi.com/article/10.3390/ijms27167289/s1.

## Abbreviations

AMPA	α-amino-3-hydroxy-5-methyl-4-isoxazolepropionic acid
AMPAR	AMPA receptor
bp	Base pair
CGGA	Chinese Glioma Genome Atlas
GBM	Glioblastoma
GDC	Genome Data Commons
GEO	Gene Expression Omnibus
GO	Gene Ontology
IDH	Isocitrate dehydrogenase
LGG	Lower-grade glioma
LGG-IDH-mut-codel	Lower-grade glioma harboring IDH mutation with 1p/19q co-deletion
LGG-IDH-mut-non-codel	Lower-grade glioma harboring IDH mutation lacking 1p/19q co-deletion
LGG-IDH-wt	Lower-grade glioma IDH-wild-type
LTP	Long-term potentiation
MAGUK	Membrane-associated guanylate kinase
MAPK	Mitogen-activated protein kinase
MRG	Mitochondrial-related gene
MRM	Mitochondrial RNA modification
mtDNA	Mitochondrial DNA
mTOR	Mammalian target of rapamycin
mtPCG	Mitochondrial protein-coding gene
OS	Overall survival
OXPHOS	Oxidative phosphorylation
PI3K	Phosphoinositide 3-kinase
ROS	Reactive oxygen species
SNARE	Soluble *N*-ethylmaleimide-sensitive factor Attachment protein Receptor
TCGA	The Cancer Genome Atlas
WHO	World Health Organization
