# Supplementary material for: A Coordinated Mitochondrial Genome Expression Program Marks a Differentiated Neural-Lineage and Synaptic Transcriptional State in Lower-Grade Glioma
Source: Int J Mol Sci. 2026 Aug 15;27(16):7289. doi: 10.3390/ijms27167289 (PMC13513048; doi:10.3390/ijms27167289)
Supplement: Supplementary file 1 [file ijms-27-07289-s001.zip › ijms-4369107-supplementary.pdf]

Supplementary Information for

A Coordinated Mitochondrial Genome Expression Program Marks a Differentiated Neural-Lineage and Synaptic Transcriptional State in Lower-Grade Glioma

Henrique Ritter Dal-Pizzol, Mariane Jaeger, Caroline Brunetto de Farias, André T. Brunetto, Gustavo R. Isolan, and Rafael Roesler

Supplementary Table S1. Correlations among expression levels of mtPCGs in TCGA LGG tumors.

**GENE**	*MT-ND1*	*MT-ND2*	*MT-CO1*	*MT-CO2*	*MT-ATP8*	*MT-ATP6*	*MT-CO3*	*MT-ND3*	*MT-ND4L*	*MT-ND4*	*MT-ND5*	*MT-ND6*	*MT-CYB*
MT-ND1	1	+++ 0.92	++ 0.73	++ 0.65	+ 0.53	++ 0.80	++ 0.75	++ 0.67	+ 0.46	++ 0.75	+ 0.61	+ 0.54	++ 0.79
MT-ND2	+++ 0.92	1	++ 0.68	++ 0.65	+ 0.47	++ 0.83	++ 0.68	++ 0.74	NC 0.43	++ 0.78	++ 0.62	+ 0.57	++ 0.77
MT-CO1	++ 0.73	++ 0.68	1	++ 0.83	NC 0.43	++ 0.74	++ 0.84	+ 0.59	+ 0.56	++ 0.80	++ 0.77	++ 0.62	++ 0.77
MT-CO2	++ 0.65	++ 0.65	++ 0.83	1	NC 0.42	++ 0.78	++ 0.85	++ 0.72	NC 0.43	++ 0.79	+ 0.58	+ 0.60	++ 0.78
MT-ATP8	+ 0.53	+ 0.47	NC 0.43	NC 0.42	1	++ 0.62	NC 0.44	NC 0.42	++ 0.64	+ 0.61	+ 0.53	NC 0.41	++ 0.63
MT-ATP6	++ 0.80	++ 0.83	++ 0.74	++ 0.78	++ 0.62	1	++ 0.76	++ 0.79	NC 0.39	++ 0.88	++ 0.64	++ 0.73	++ 0.85
MT-CO3	++ 0.75	++ 0.68	++ 0.84	++ 0.85	NC 0.44	++ 0.76	1	++ 0.68	NC 0.46	++ 0.73	+ 0.51	NC 0.46	++ 0.78
MT-ND3	++ 0.67	++ 0.74	+ 0.59	++ 0.72	NC 0.42	++ 0.79	++ 0.68	1	NC 0.35	++ 0.73	+ 0.50	+ 0.61	++ 0.75
MT-ND4L	NC 0.46	NC 0.43	+ 0.56	NC 0.43	++ 0.64	NC 0.39	+ 0.46	NC 0.35	1	++ 0.63	++ 0.67	NC 0.18	+ 0.53
MT-ND4	++ 0.75	++ 0.78	++ 0.80	++ 0.79	+ 0.61	++ 0.88	++ 0.73	++ 0.73	++ 0.63	1	++ 0.77	++ 0.69	++ 0.86
MT-ND5	+ 0.61	++ 0.62	++ 0.77	++ 0.58	++ 0.53	++ 0.64	+ 0.51	+ 0.50	++ 0.67	++ 0.77	1	++ 0.77	++ 0.73
MT-ND6	+ 0.54	+ 0.57	++ 0.62	+ 0.60	NC 0.41	++ 0.73	NC 0.46	+ 0.61	NC 0.18	++ 0.69	++ 0.77	1	++ 0.67
MT-CYB	++ 0.79	++ 0.77	++ 0.77	++ 0.78	++ 0.63	++ 0.85	++ 0.78	++ 0.75	+ 0.53	++ 0.86	++ 0.73	++ 0.67	1

mtPCGs are shown in genomic order. Positive moderate correlations (+) were defined as Pearson's r between 0.45 and 0.61, strong correlations (++) as r between 0.62 and 0.88, and very strong (+++) correlations as $r > 0.89$ with a statistically significant p value; NC, no correlation defined by $r < 0.45$. Analyses were carried out using the Evergene platform [49] and are shown in Supplementary Figure S7.

Supplementary Table S2. Cut-off values for high and low gene expression levels used for survival analyses.

Dataset	Gene	Cutpoint	Low expression (*n*)	High expression (*n*)
TCGA-LGG	*MT-ND1*	0.846	98	392
	MT-ND2	0.644	118	372
	MT-CO1	0.825	101	389
	MT-CO2	-0.754	101	389
	MT-ATP8	-0.395	157	333
	MT-ATP6	-0.693	112	378
	MT-CO3	-0.581	125	365
	MT-ND3	-0.607	123	367
	MT-ND4L	-0.243	178	312
	MT-ND4	-0.719	110	380
	MT-ND5	0.115	254	236
	MT-ND6	0.090	272	218
	MT-CYB	-0.377	169	321
CGGA LGG	*MT-ND1*	-0.172	121	286
	MT-ND2	-0.243	101	306
	MT-CO1	0.674	325	82
	MT-CO2	-0.147	120	287
	MT-ATP8	1.124	315	92
	MT-ATP6	-0.119	106	301
	MT-CO3	-0.026	136	271
	MT-ND3	-0.229	91	316
	MT-ND4L	0.628	273	134
	MT-ND4	-0.133	134	273
	MT-ND5	0.596	271	136
	MT-ND6	-0.681	81	326
	MT-CYB	-0.431	81	326

All expression values were standardized using gene-wise z-score transformation.

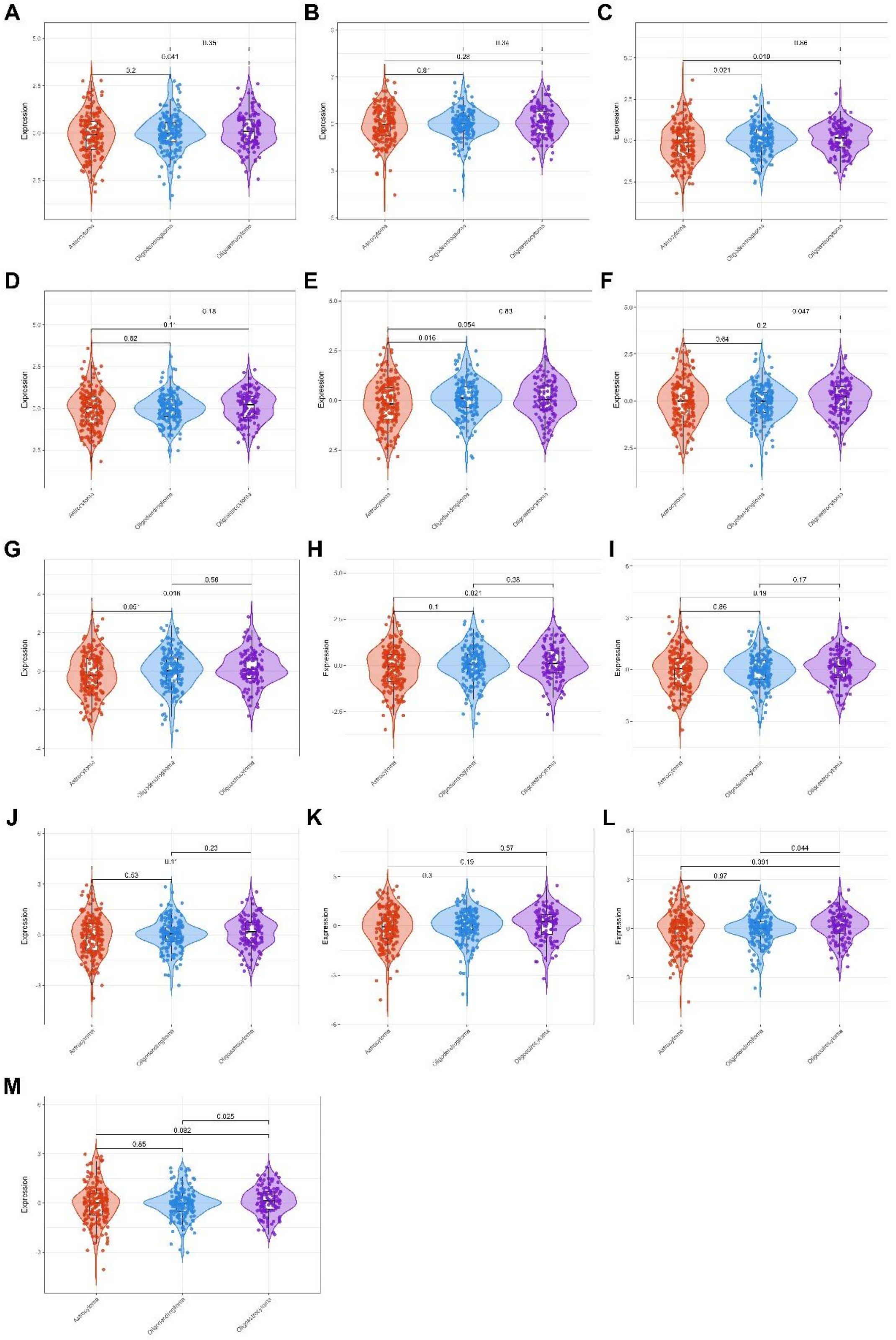

Supplementary Figure S1. Gene expression levels of mtPCGs in TCGA LGG tumors classified into histological types. (**A**) *MT-ND1;* (**B**) *MT-ND2;* (**C**) *MT-CO1;* (**D**) *MT-CO2;* (**E**) *MT-ATP8;* (**F**) *MT-ATP6;* (**G**) *MT-CO3;* (**H**) *MT-ND3;* (**I**) *MT-ND4L;* (**J**) *MT-ND4*; (**K**) *MT-ND5*; (**L**) *MT-ND6*; (**M**) *MT-CYB*. Astrocytoma, n = 188; oligodendroglioma, n = 179; oligoastrocytoma, n = 123; adjusted p-values are indicated in the panels.

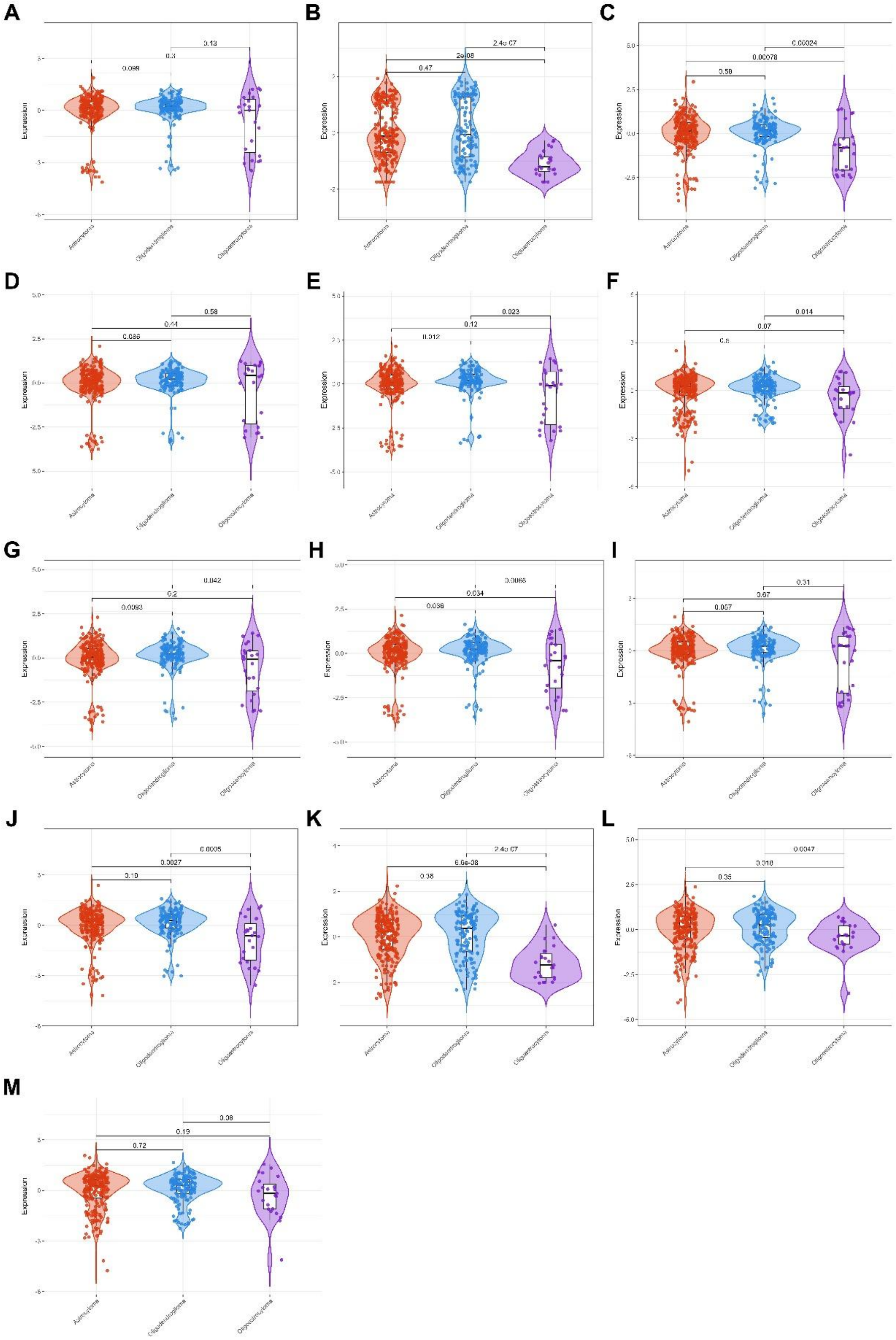

Supplementary Figure S2. Gene expression levels of mtPCGs in CGGA LGG tumors classified into histological types. (**A**) *MT-ND1;* (**B**) *MT-ND2;* (**C**) *MT-CO1;* (**D**) *MT-CO2;* (**E**) *MT-ATP8;* (**F**) *MT-ATP6;* (**G**) *MT-CO3;* (**H**) *MT-ND3;* (**I**) *MT-ND4L;* (**J**) *MT-ND4*; (**K**) *MT-ND5*; (**L**) *MT-ND6*; (**M**) *MT-CYB*. Astrocytoma, $n = 239$; oligodendroglioma, $n = 145$; oligoastrocytoma, $n = 23$; adjusted *P*-values are indicated in the panels.

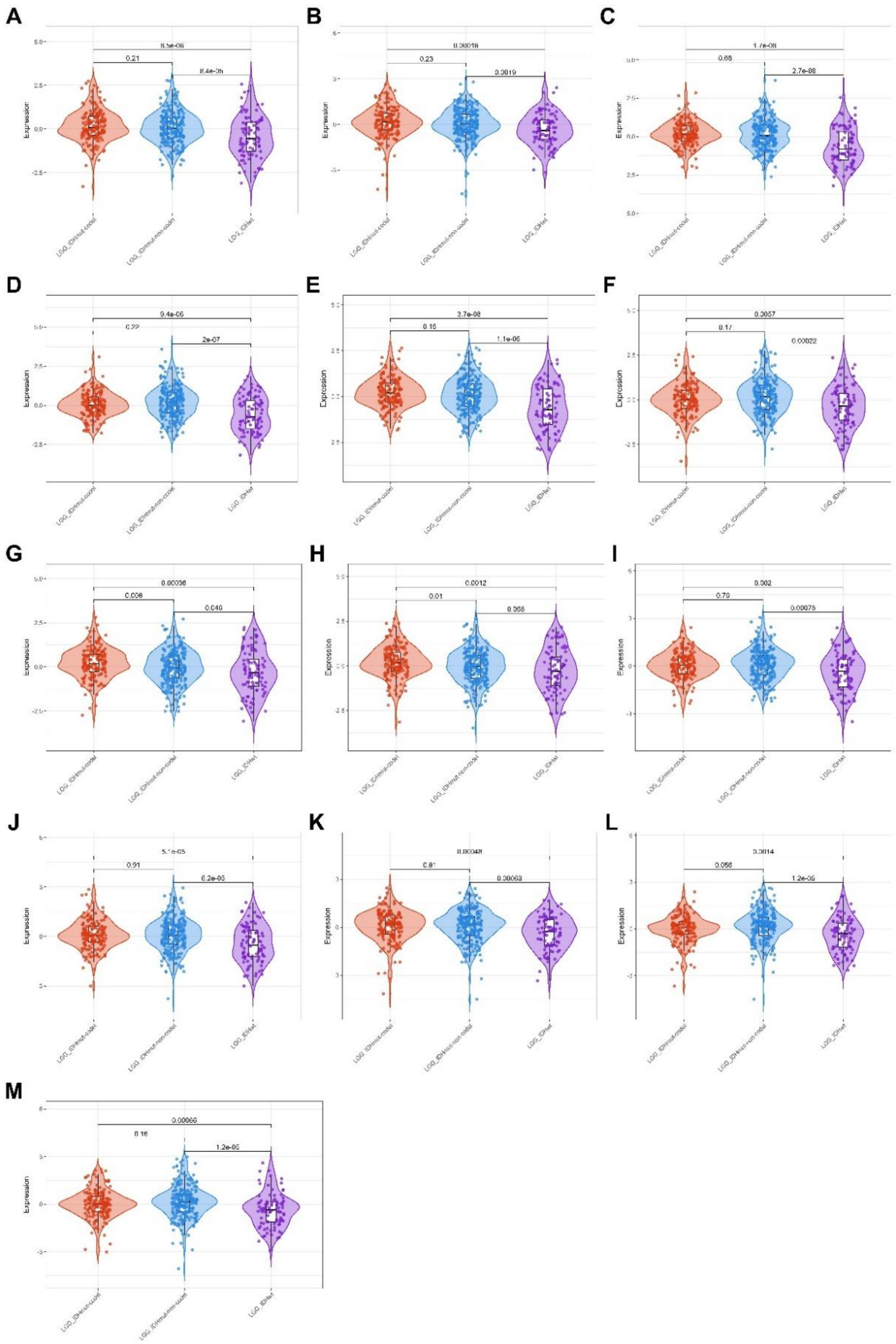

Supplementary Figure S3. Gene expression levels of mtPCGs in CGGA LGG tumors classified into molecular subtypes. (**A**) *MT-ND1*; (**B**) *MT-ND2*; (**C**) *MT-CO1*; (**D**) *MT-CO2*; (**E**) *MT-ATP8*; (**F**) *MT-ATP6*; (**G**) *MT-CO3*; (**H**) *MT-ND3*; (**I**) *MT-ND4L*; (**J**) *MT-ND4*; (**K**) *MT-ND5*; (**L**) *MT-ND6*; (**M**) *MT-CYB*. LGG-IDH-mut-codel, n = 113, LGG-IDH-mut-non-codel, n = 142, LGG-IDH-wt, n = 88; p-values are indicated in the panels.

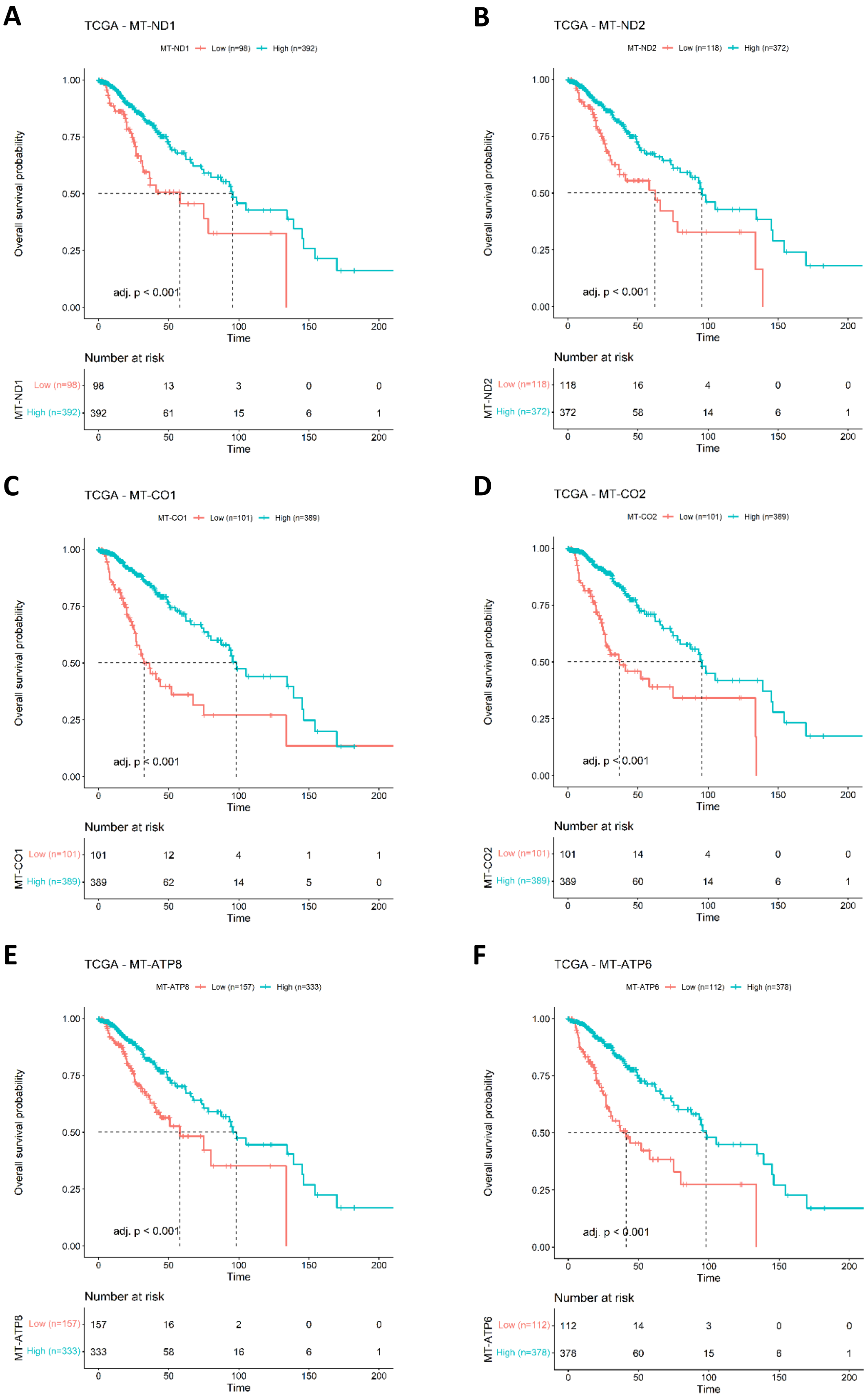
A
TCGA - MT-ND1
MT-ND1 Low (n=98) High (n=392)
Overall survival probability
adj. p < 0.001
Time
Number at risk
Low (n=98) 98 13 3 0 0
High (n=392) 392 61 15 6 1
B
TCGA - MT-ND2
MT-ND2 Low (n=118) High (n=372)
Overall survival probability
adj. p < 0.001
Time
Number at risk
Low (n=118) 118 16 4 0 0
High (n=372) 372 58 14 6 1
C
TCGA - MT-CO1
MT-CO1 Low (n=101) High (n=389)
Overall survival probability
adj. p < 0.001
Time
Number at risk
Low (n=101) 101 12 4 1 1
High (n=389) 389 62 14 5 0
D
TCGA - MT-CO2
MT-CO2 Low (n=101) High (n=389)
Overall survival probability
adj. p < 0.001
Time
Number at risk
Low (n=101) 101 14 4 0 0
High (n=389) 389 60 14 6 1
E
TCGA - MT-ATP8
MT-ATP8 Low (n=157) High (n=333)
Overall survival probability
adj. p < 0.001
Time
Number at risk
Low (n=157) 157 16 2 0 0
High (n=333) 333 58 16 6 1
F
TCGA - MT-ATP6
MT-ATP6 Low (n=112) High (n=378)
Overall survival probability
adj. p < 0.001
Time
Number at risk
Low (n=112) 112 14 3 0 0
High (n=378) 378 60 15 6 1

G

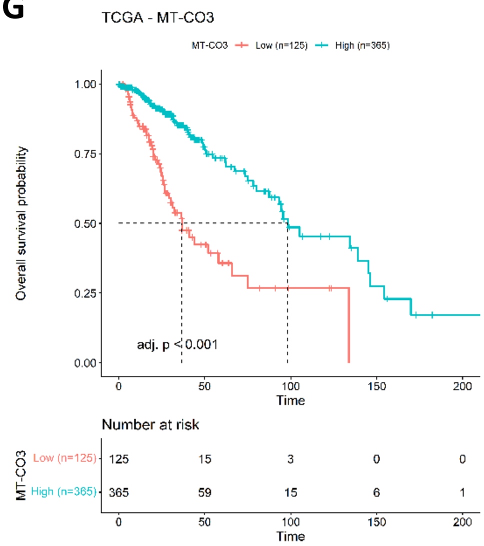
TCGA - MT-CO3
MT-CO3 Low (n=125) High (n=365)
Overall survival probability
1.00
0.75
0.50
0.25
0.00
adj. p < 0.001
0 50 100 150 200
Time
Number at risk
MT-CO3
Low (n=125) 125 15 3 0 0
High (n=365) 365 59 15 6 1
0 50 100 150 200
Time

H

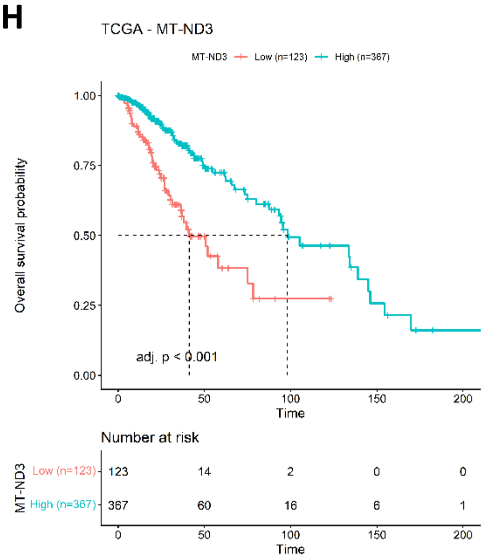
TCGA - MT-ND3
MT-ND3 Low (n=123) High (n=367)
Overall survival probability
1.00
0.75
0.50
0.25
0.00
adj. p < 0.001
0 50 100 150 200
Time
Number at risk
MT-ND3
Low (n=123) 123 14 2 0 0
High (n=367) 367 60 16 6 1
0 50 100 150 200
Time

I

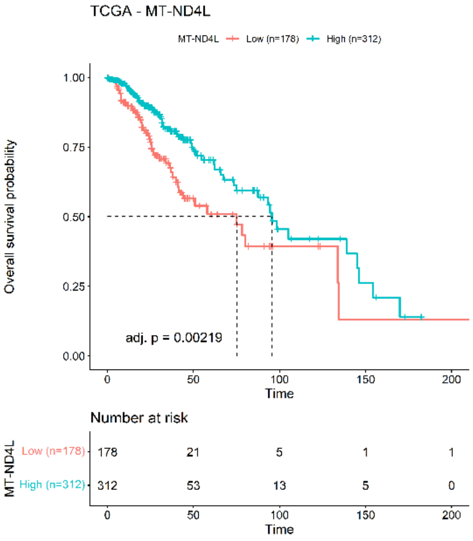
TCGA - MT-ND4L
MT-ND4L Low (n=178) High (n=312)
Overall survival probability
1.00
0.75
0.50
0.25
0.00
adj. p = 0.00219
0 50 100 150 200
Time
Number at risk
MT-ND4L
Low (n=178) 178 21 5 1 1
High (n=312) 312 53 13 5 0
0 50 100 150 200
Time

J

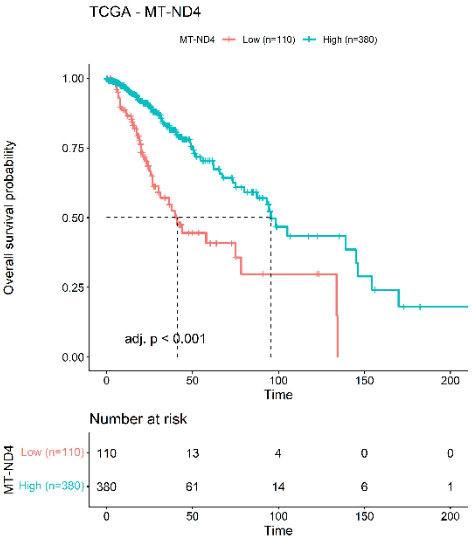
TCGA - MT-ND4
MT-ND4 Low (n=110) High (n=380)
Overall survival probability
1.00
0.75
0.50
0.25
0.00
adj. p < 0.001
0 50 100 150 200
Time
Number at risk
MT-ND4
Low (n=110) 110 13 4 0 0
High (n=380) 380 61 14 6 1
0 50 100 150 200
Time

K

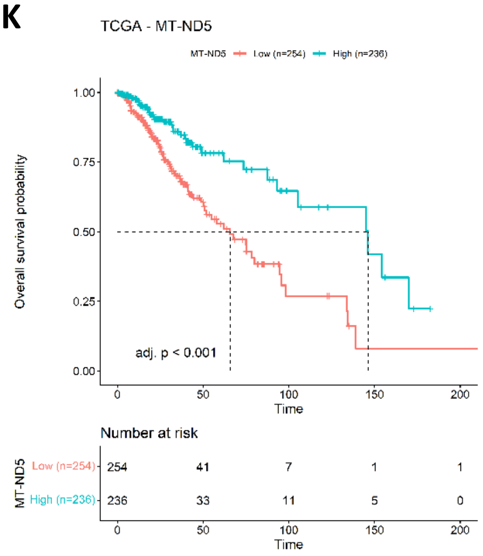
TCGA - MT-ND5
MT-ND5 Low (n=254) High (n=236)
Overall survival probability
1.00
0.75
0.50
0.25
0.00
adj. p < 0.001
0 50 100 150 200
Time
Number at risk
MT-ND5
Low (n=254) 254 41 7 1 1
High (n=236) 236 33 11 5 0
0 50 100 150 200
Time

L

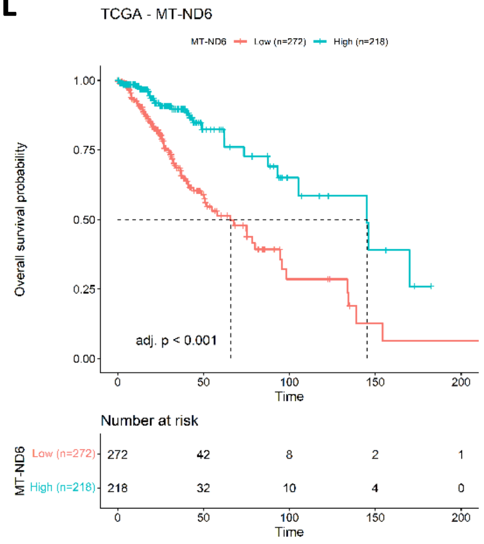
TCGA - MT-ND6
MT-ND6 Low (n=272) High (n=218)
Overall survival probability
1.00
0.75
0.50
0.25
0.00
adj. p < 0.001
0 50 100 150 200
Time
Number at risk
MT-ND6
Low (n=272) 272 42 8 2 1
High (n=218) 218 32 10 4 0
0 50 100 150 200
Time

M

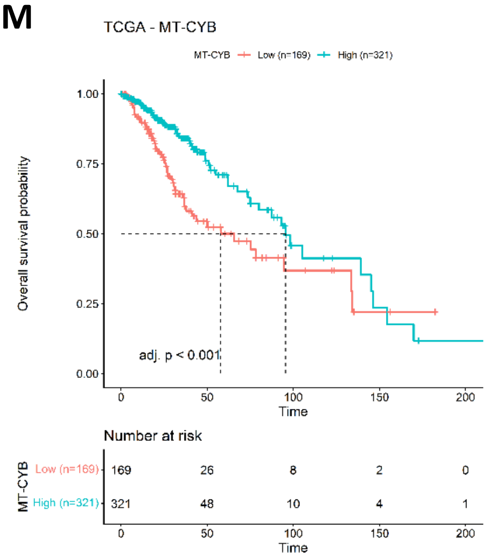

Supplementary Figure S4. High expression of mtPCGs is associated with longer survival in patients with TCGA LGG tumors. (**A**) *MT-ND1*; (**B**) *MT-ND2*; (**C**) *MT-CO1*; (**D**) *MT-CO2*; (**E**) *MT-ATP8*; (**F**) *MT-ATP6*; (**G**) *MT-CO3*; (**H**) *MT-ND3*; (**I**) *MT-ND4L*; (**J**) *MT-ND4*; (**K**) *MT-ND5*; (**L**) *MT-ND6*; (**M**) *MT-CYB.* The number of samples and adjusted *p*-values are indicated in the panels.

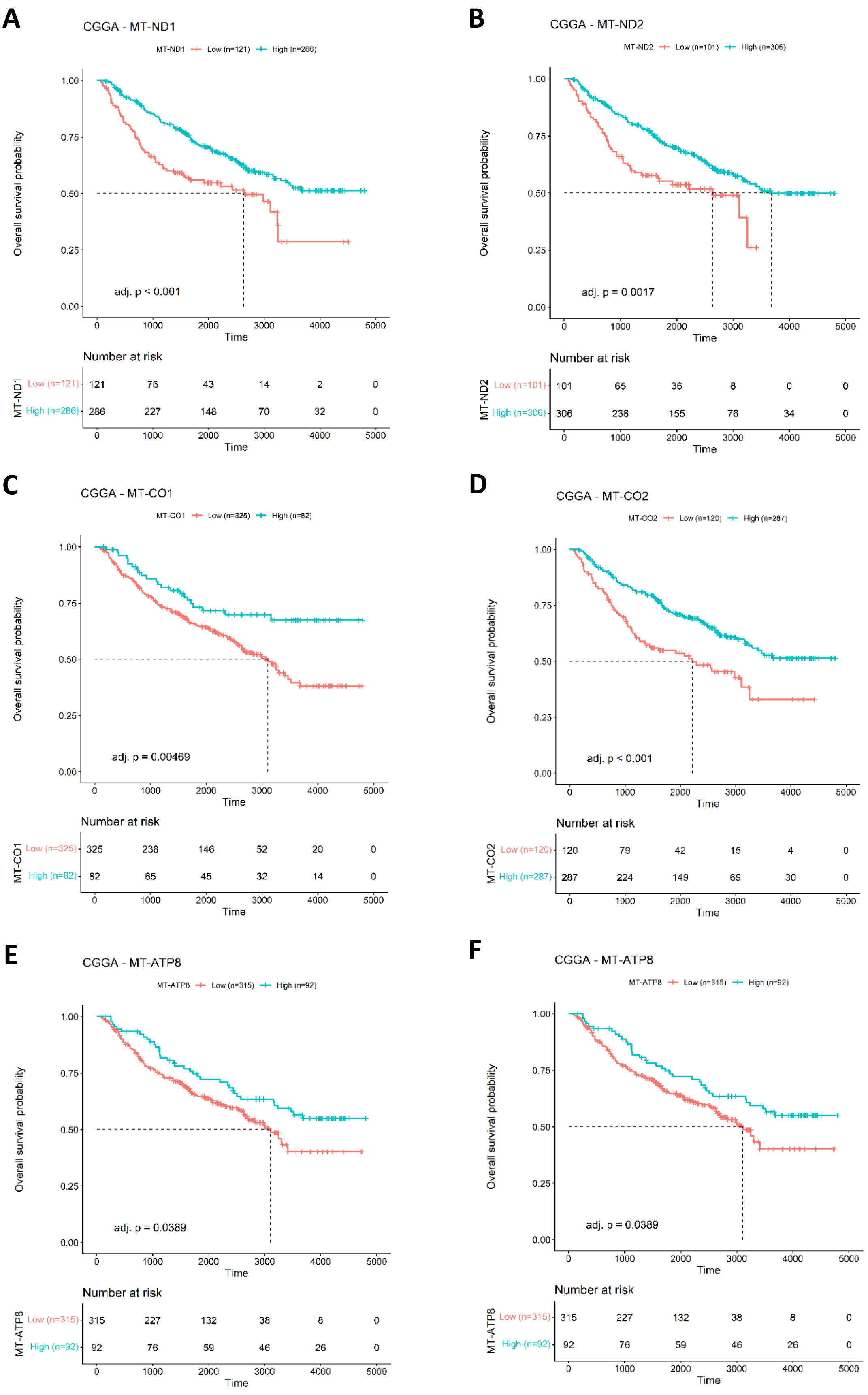
A
CGGA - MT-ND1
MT-ND1 Low (n=121) High (n=286)
Overall survival probability
1.00
0.75
0.50
0.25
0.00
adj. p < 0.001
0 1000 2000 3000 4000 5000
Time
Number at risk
MT-ND1
Low (n=121) 121 76 43 14 2 0
High (n=286) 286 227 148 70 32 0
B
CGGA - MT-ND2
MT-ND2 Low (n=101) High (n=306)
adj. p = 0.0017
Number at risk
MT-ND2
Low (n=101) 101 65 36 8 0 0
High (n=306) 306 238 155 76 34 0
C
CGGA - MT-CO1
MT-CO1 Low (n=325) High (n=82)
adj. p = 0.00469
Number at risk
MT-CO1
Low (n=325) 325 238 146 52 20 0
High (n=82) 82 65 45 32 14 0
D
CGGA - MT-CO2
MT-CO2 Low (n=120) High (n=287)
adj. p < 0.001
Number at risk
MT-CO2
Low (n=120) 120 79 42 15 4 0
High (n=287) 287 224 149 69 30 0
E
CGGA - MT-ATP8
MT-ATP8 Low (n=315) High (n=92)
adj. p = 0.0389
Number at risk
MT-ATP8
Low (n=315) 315 227 132 38 8 0
High (n=92) 92 76 59 46 26 0
F
CGGA - MT-ATP8
MT-ATP8 Low (n=315) High (n=92)
adj. p = 0.0389
Number at risk
MT-ATP8
Low (n=315) 315 227 132 38 8 0
High (n=92) 92 76 59 46 26 0

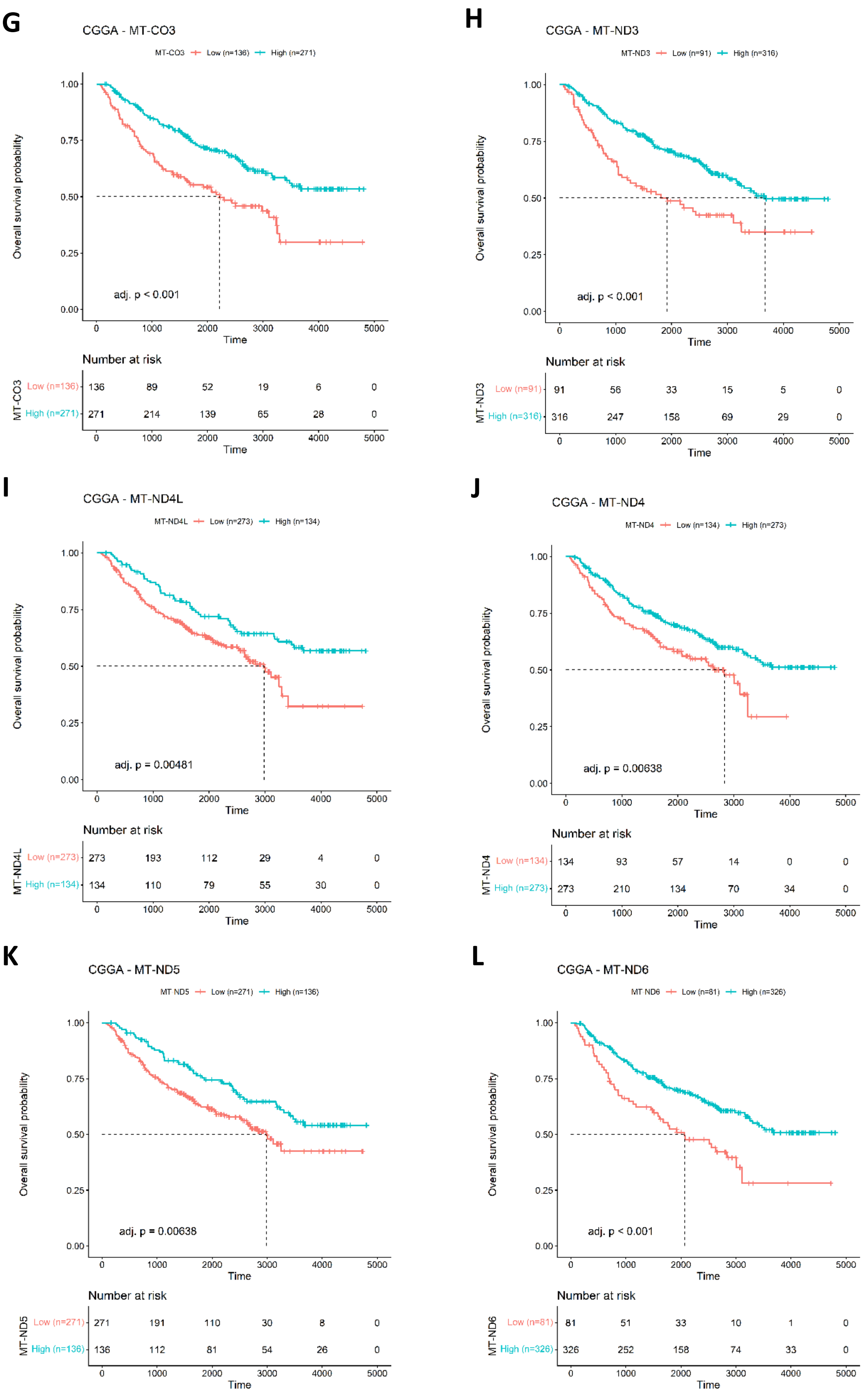
G
CGGA - MT-CO3
MT-CO3 Low (n=136) High (n=271)
Overall survival probability
adj. p < 0.001
Time
Number at risk
MT-CO3
Low (n=136) 136 89 52 19 6 0
High (n=271) 271 214 139 65 28 0
H
CGGA - MT-ND3
MT-ND3 Low (n=91) High (n=316)
adj. p < 0.001
MT-ND3
Low (n=91) 91 56 33 15 5 0
High (n=316) 316 247 158 69 29 0
I
CGGA - MT-ND4L
MT-ND4L Low (n=273) High (n=134)
adj. p = 0.00481
MT-ND4L
Low (n=273) 273 193 112 29 4 0
High (n=134) 134 110 79 55 30 0
J
CGGA - MT-ND4
MT-ND4 Low (n=134) High (n=273)
adj. p = 0.00638
MT-ND4
Low (n=134) 134 93 57 14 0 0
High (n=273) 273 210 134 70 34 0
K
CGGA - MT-ND5
MT ND5 Low (n=271) High (n=136)
adj. p = 0.00638
MT-ND5
Low (n=271) 271 191 110 30 8 0
High (n=136) 136 112 81 54 26 0
L
CGGA - MT-ND6
MT ND6 Low (n=81) High (n=326)
adj. p < 0.001
MT-ND6
Low (n=81) 81 51 33 10 1 0
High (n=326) 326 252 158 74 33 0

M

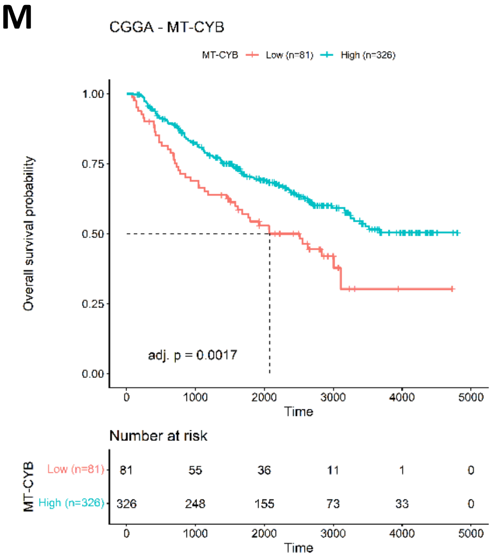

Supplementary Figure S5. High expression of mtPCGs is associated with longer survival in patients with CGGA LGG tumors. (**A**) *MT-ND1*; (**B**) *MT-ND2*; (**C**) *MT-CO1*; (**D**) *MT-CO2*; (**E**) *MT-ATP8*; (**F**) *MT-ATP6*; (**G**) *MT-CO3*; (**H**) *MT-ND3*; (**I**) *MT-ND4L*; (**J**) *MT-ND4*; (**K**) *MT-ND5*; (**L**) *MT-ND6*; (**M**) *MT-CYB*. The number of samples and adjusted *p*-values are indicated in the panels.

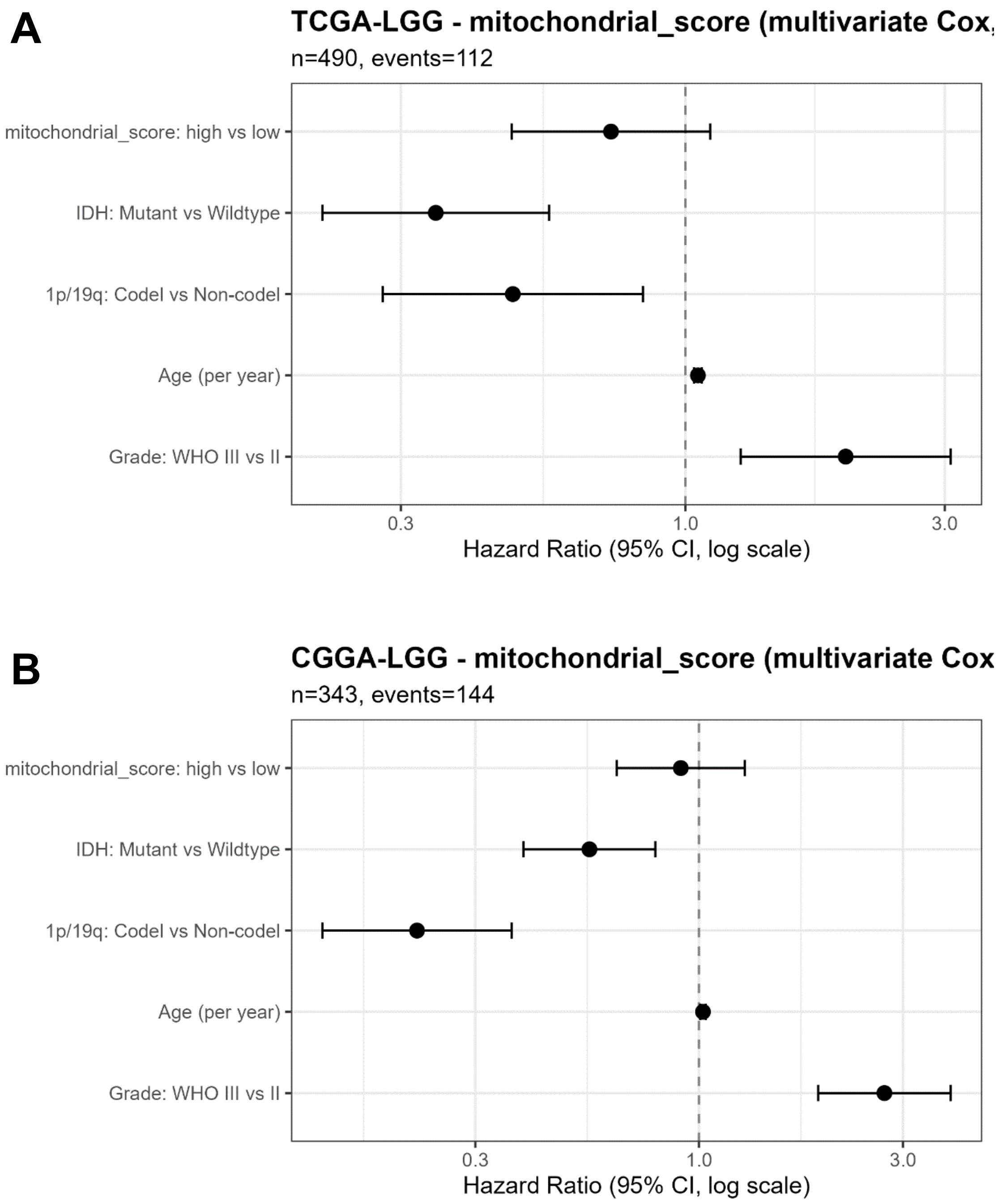

Supplementary Figure S6. Forest plots showing multivariable Cox proportional hazards analyses for OS in (**A**) TCGA and (**B**) CGGA LLG tumors. Models included the mitochondrial gene score composed by the combined 13 mtPCGs together with established clinicomolecular prognostic variables, namely IDH mutation status, 1p/19q codeletion status, age, and tumor grade. Hazard ratios (HRs) and 95% confidence intervals are shown. HR < 1 indicates favorable prognostic association, whereas HR > 1 indicates increased risk of death.

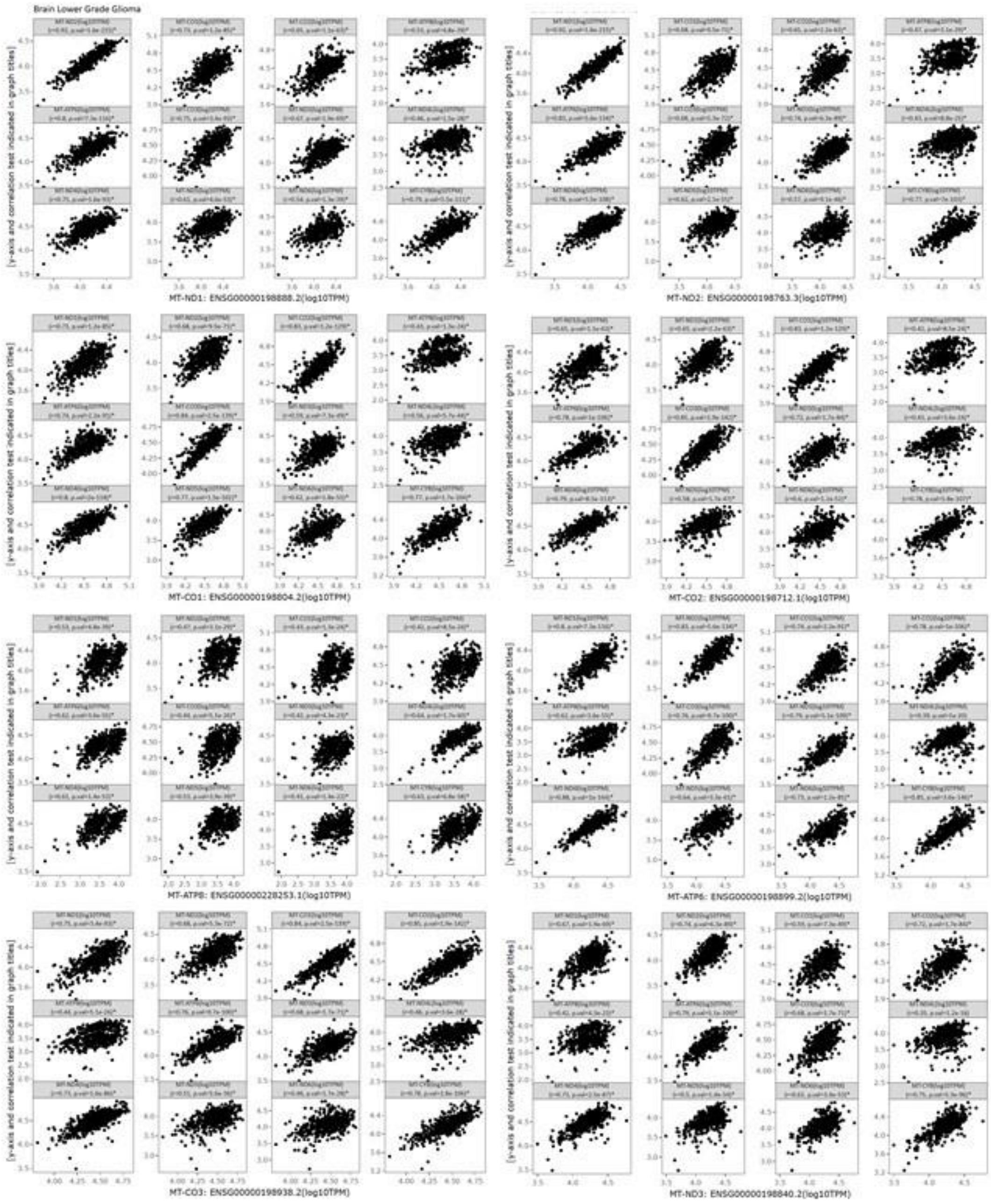
Brain Lower Grade Glioma
[y-axis and correlation test indicated in graph titles]
MT-ND1: ENSG00000198888.2(log10TPM)
MT-ND2: ENSG00000198763.3(log10TPM)
MT-CO1: ENSG00000198804.2(log10TPM)
MT-CO2: ENSG00000198712.1(log10TPM)
MT-ATP8: ENSG00000228253.1(log10TPM)
MT-ATP6: ENSG00000198899.2(log10TPM)
MT-CO3: ENSG00000198938.2(log10TPM)
MT-ND3: ENSG00000198840.2(log10TPM)

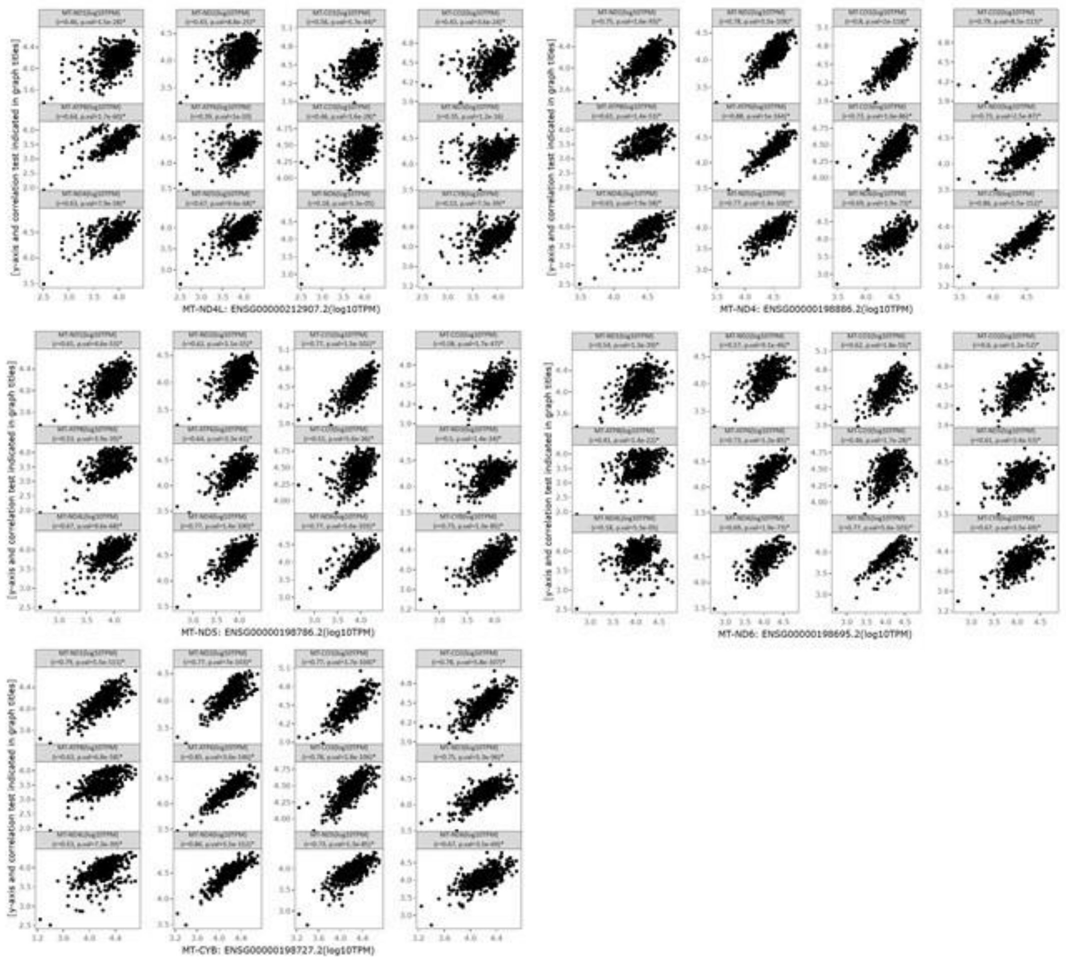

Supplementary Figure S7. Correlations found among expression of mtPCGs in TCGA LGG tumors; Pearson's *r* and *p* values are indicated in the panels. Analyses were carried out using the Evergene platform [49].

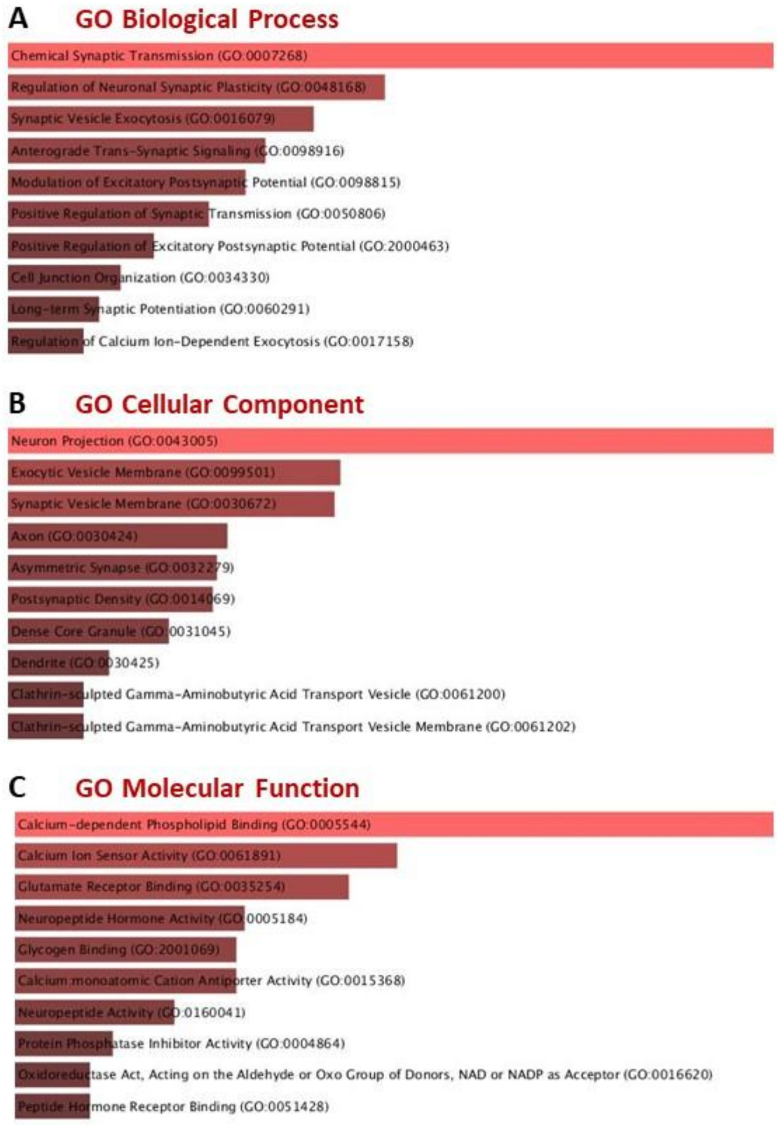

Supplementary Figure S8. Gene Ontology (GO) enrichment analysis of genes associated with the mitochondrial score in LGG. Transcriptome-wide Spearman correlation analysis was performed between the mitochondrial score and all nuclear genes in the TCGA-LGG cohort. The 200 genes most strongly positively correlated with the mitochondrial score were subjected to GO enrichment analysis using Enrichr. The top enriched terms are shown for (**A**) Biological Process, (**B**) Cellular Component, and (**C**) Molecular Function categories. Enriched terms were predominantly related to neuronal differentiation, synaptic signaling, synaptic organization, ion channel activity, and neuronal cellular compartments, supporting an association between the mitochondrial transcriptional program and a neural/synaptic gene expression signature in LGG. Analyses were performed in Enrich [50].
